# Herpes Simplex Virus 2 *ICP0^−^* Mutant Viruses Are Avirulent and Immunogenic: Implications for a Genital Herpes Vaccine

**DOI:** 10.1371/journal.pone.0012251

**Published:** 2010-08-17

**Authors:** William P. Halford, Ringo Püschel, Brandon Rakowski

**Affiliations:** Department of Microbiology and Immunology, Southern Illinois University School of Medicine, Springfield, Illinois, United States of America; Institute of Infectious Disease and Molecular Medicine, South Africa

## Abstract

Herpes simplex virus 1 (HSV-1) *ICP0* ^−^ mutants are interferon-sensitive, avirulent, and elicit protective immunity against HSV-1 (*Virol J*, 2006, 3:44). If an *ICP0* ^−^ mutant of herpes simplex virus 2 (HSV-2) exhibited similar properties, such a virus might be used to vaccinate against genital herpes. The current study was initiated to explore this possibility. Several HSV-2 *ICP0* ^−^ mutant viruses were constructed and evaluated in terms of three parameters: ***i.*** interferon-sensitivity; ***ii.*** virulence in mice; and ***iii.*** capacity to elicit protective immunity against HSV-2. One *ICP0* ^−^ mutant virus in particular, HSV-2 0ΔNLS, achieved an optimal balance between avirulence and immunogenicity. HSV-2 0ΔNLS was interferon-sensitive in cultured cells. HSV-2 0ΔNLS replicated to low levels in the eyes of inoculated mice, but was rapidly repressed by an innate, Stat 1-dependent host immune response. HSV-2 0ΔNLS failed to spread from sites of inoculation, and hence produced only inapparent infections. Mice inoculated with HSV-2 0ΔNLS consistently mounted an HSV-specific IgG antibody response, and were consistently protected against lethal challenge with wild-type HSV-2. Based on their avirulence and immunogenicity, we propose that HSV-2 *ICP0* ^−^ mutant viruses merit consideration for their potential to prevent the spread of HSV-2 and genital herpes.

## Introduction

Herpes simplex virus 1 (HSV-1) or herpes simplex virus 2 (HSV-2) may cause primary genital herpes [1], [2], [3]. In contrast, more than 90% of cases of recurrent genital herpes are caused by HSV-2 [1], [4]. Recurrent genital herpes is a physically superficial disease in most cases, but often produces emotionally debilitating effects [5], [6], [7]. Because HSV-2 is responsible for the bulk of disease, an effective HSV-2 vaccine is sought that may stop the spread of genital herpes.

HSV-1 and HSV-2 are similar viruses that share a nearly identical set of ∼75 co-linear genes distributed across 152 and 154 kbp dsDNA genomes, respectively. HSV-1 and HSV-2 establish life-long infections in their human hosts, and both viruses persist by establishing latent infections in the human nervous system. Infections with these viruses are exceedingly common; HSV-1 infects ∼4 billion people and HSV-2 infects ∼1 billion people [8]. Approximately 5% of HSV-2 infected individuals live with genital herpes disease that recurs once every 3 to 12 months [9], [10]. HSV-2 infections spread to new individuals at a rate of ∼20 million per year. An effective HSV-2 vaccine would be useful in breaking this cycle, and protecting young adults from the 1 in 10 chance that they will acquire HSV-2 before they marry [4], [11], [12].

For decades, efforts to develop an HSV-2 vaccine have been predicated on the assumption that a live-attenuated HSV-2 virus would be too risky for use as a human vaccine, and safer alternatives have been sought [13], [14]. The non-replicating HSV-2 vaccines that have been most seriously considered are ***1.*** HSV-2 subunit vaccines and ***2.*** replication-defective HSV-2 viruses. The immunodominant HSV-2 glycoprotein D (gD_2_) antigen has been considered for its potential to serve as a subunit vaccine. After more than a decade of testing in human clinical trials, it remains unclear that vaccination with a gD_2_ subunit renders human recipients immune to wild-type HSV-2 infections and/or genital herpes [15], [16]. One of the limitations of an HSV-2 subunit vaccine is that vaccine recipients are only exposed to 1% of HSV-2's antigens (i.e., 1 of ∼80 proteins). Replication-defective HSV-2 viruses offer the advantage that nearly all of HSV-2's antigens may be expressed at the site of inoculation and presented to CD8^+^ T cells in the context of the MHC class I pathway [17], [18]. However, it remains unclear if a replication-defective HSV-2 virus may recapitulate the *magnitude* and *duration* to the immune response that is elicited against a live, replicating virus such as wild-type HSV-2.

An HSV-2 vaccine should not only be safe, but it should also be effective. For decades, live HSV-2 viruses have been largely excluded from consideration as a genital herpes vaccine on the grounds that a live-attenuated HSV-2 vaccine would be too dangerous. However, this safety-based rationale is incongruous with the 200-year history of viral vaccines. Approximately 75% of the vaccines that have succeeded in preventing human viral disease have contained live, replicating viruses. Pediatricians and parents have deemed the approach safe enough for the past 50 years to warrant the inoculation of hundreds of millions of children with live, replicating viruses, and millions of human lives have been spared from death or disfigurement as a result.

Historically, live-attenuated viruses have been our most effective mode of vaccination. Originally, the word ‘*vaccination*’ specifically meant to inject a person with live, replicating *vaccinia* virus in order to elicit a cross-protective response that provided immunity against the smallpox virus [19]. Most of our effective viral vaccines emulate the original approach, and rely on inoculation of humans with live viruses that establish mild or inapparent infections that cross-protect against their more virulent counterparts that exist in nature. Such live-attenuated viruses are the active ingredient in the oral poliovirus vaccine, the MMR (mumps, measles, rubella) vaccine, and the chickenpox and shingles vaccine [20], [21], [22]. Isolated reports have raised the possibility that a live-attenuated HSV-2 vaccine might be feasible [23], [24], [25]. However, a live-attenuated HSV-2 vaccine has not been systematically investigated due to concerns surrounding the safety of administering a live α-herpesvirus to millions of people [26].

Tens of millions of children have now been inoculated with the live-attenuated Oka strain of varicella-zoster virus (VZV) [27]. Like HSV-1 and HSV-2, VZV is an α-herpesvirus that routinely establishes life-long infections in the human nervous system [28]. The live VZV vaccine has proven safe and effective in preventing the epidemic spread of chickenpox [20], [27], and is now being used to prevent the age-onset disease of ‘shingles’ caused by reactivated VZV infections [29], [30], [31]. The success of the chickenpox vaccine demonstrates that a live and appropriately attenuated α-herpesvirus may be used to safely control a human disease. If this principle may be expanded to include HSV-1 and HSV-2, then genital herpes might also be prevented using a live-attenuated HSV-2 virus as a vaccine. The feasibility of this proposal remains unclear because it has not been investigated.

The *ICP0* gene encodes a critical regulatory protein, infected cell protein 0 (ICP0), which controls HSV-1's balance between latency and replication (reviewed in Ref. [32], [33], [34]). HSV-1 viruses that bear null mutations in the *ICP0* gene are acutely sensitive to repression by interferon-α/β [35], [36], are avirulent in immunocompetent mice and lymphocyte-deficient *rag2* ^−/−^ mice [37], and elicit a nearly sterilizing immune response against wild-type HSV-1 [37]. These studies have led us to predict that a live HSV-2 vaccine strain may be derived by mutagenesis of HSV-2's *ICP0* gene [13], [37].

The current study was initiated to test this hypothesis. Five HSV-2 *ICP0* ^−^ mutant viruses were constructed and characterized using a screening procedure designed to determine if any of these mutant viruses achieved the type of balance between safety and immunogenicity that should be expected of a live-attenuated vaccine strain. We report that one HSV-2 *ICP0* ^−^ mutant virus in particular, HSV-2 0ΔNLS, exhibited such properties. HSV-2 0ΔNLS was interferon-sensitive, avirulent in immunocompetent animals, and elicited robust protection in recipients against later exposures to wild-type HSV-2. Based on these results, we propose that interferon-sensitive HSV-2 *ICP0* ^−^ mutant viruses warrant consideration for their potential to prevent the spread of HSV-2 and genital herpes in the human population.

## Results

### Construction and characterization of HSV-2 ICP0^−^ mutant viruses

**Figure 1:**
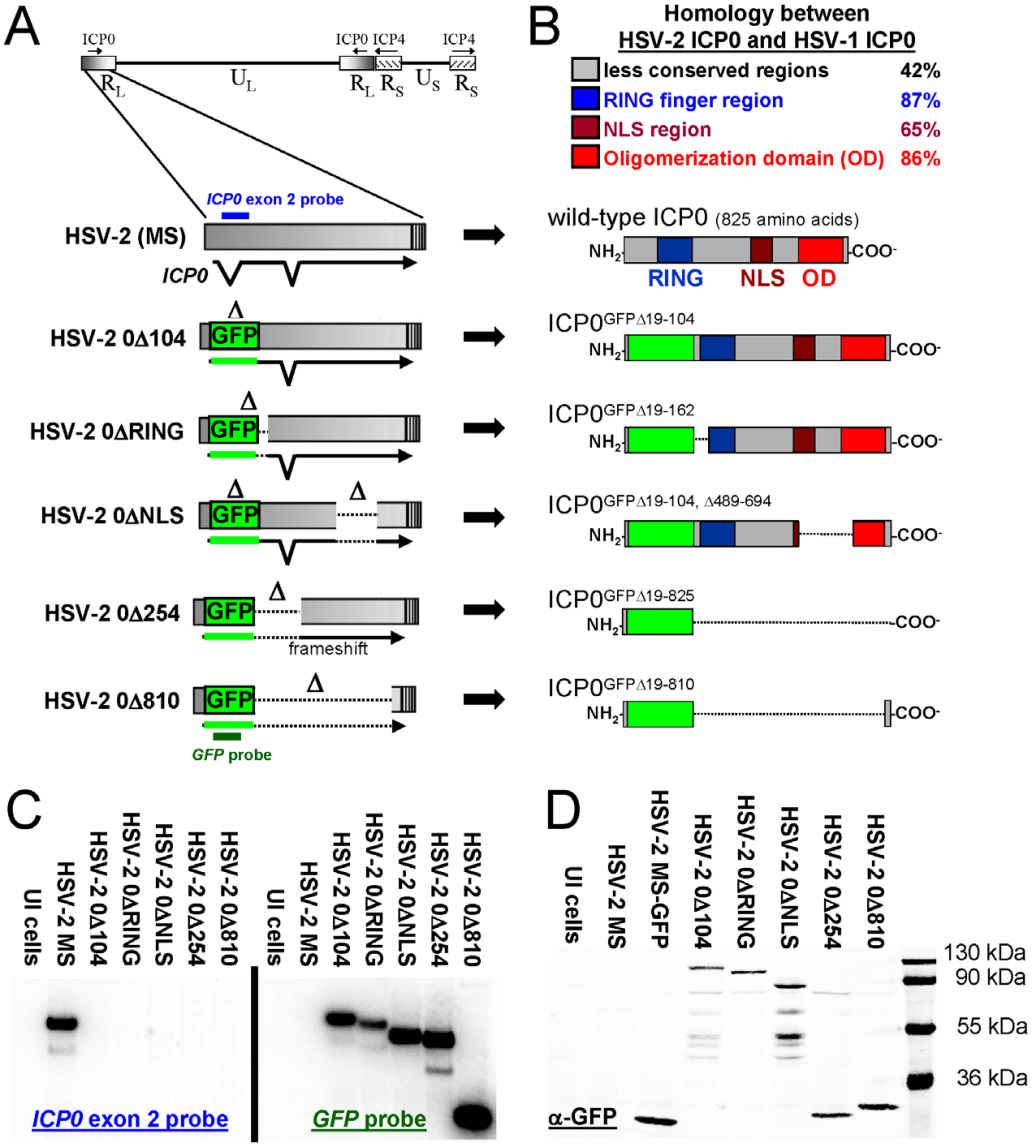
Description of HSV-2 *ICP0* ^−^ mutant viruses. (**A**) Mutations in the HSV-2 *ICP0* gene. Schematics indicate the relative size and location of a *GFP*-coding sequence that replaced codons 19–104 in all HSV-2 *ICP0* ^−^ mutants, as well as the size and location of secondary deletions in HSV-2 0ΔRING, 0ΔNLS, 0Δ254, or 0Δ810. (**B**) Regions of ICP0 conserved between HSV-1 and HSV-2 are color-coded above, and below is shown the effects of deletions on ICP0's conserved RING finger region, nuclear localization signal (NLS) region, and/or oligomerization domain. (**C**) Southern blot analysis of SacI – StuI digested DNA harvested from cells that were uninfected (UI) or were inoculated with 2.5 pfu per cell of each indicated HSV-2 virus. Replicate Southern blots were hybridized with an *ICP0* exon 2-specific oligonucleotide (shown on left) or a *GFP*-specific oligonucleotide (shown on right). The approximate locus to which the *ICP0*-specific and *GFP*-specific probes hybridized is depicted in panel A. (**D**) Western blot analysis of proteins harvested from cells that were uninfected (UI) or were inoculated with 2.5 pfu per cell of each indicated HSV-2 virus. GFP or chimeric ICP0 proteins bearing a GFP tag were labeled with rabbit polyclonal GFP-specific antibody. Molecular weight markers are shown on the right.

Three regions of ICP0 are conserved between HSV-1 and HSV-2; a RING finger-E3 ligase region [38], the region surrounding the nuclear localization signal (NLS) and adjacent phosphorylation sites [39], [40], and a C-terminal oligomerization domain [41], [42] (Fig. 1B, Fig. S1). To obtain HSV-2 viruses that varied in their level of attenuation, deletions were introduced into the *ICP0* gene that removed none, one, or all of ICP0's conserved regions (Fig. 1A, 1B). All HSV-2 *ICP0* ^−^ mutant viruses bore a common deletion that replaced codons 19 to 104 of the *ICP0* gene with a *green fluorescent protein* (*GFP*) coding sequence. HSV-2 0Δ104 contained this one mutation, and thus encoded a GFP-tagged, mutant protein that retained all of ICP0's conserved regions (Fig. 1A, 1B). A larger deletion in HSV-2 0ΔRING removed codons 19 to 162, and thus removed several cysteine residues necessary for ICP0's E3 ligase activity [43] (Fig. 1A, 1B). HSV-2 0ΔNLS contained two deletions that removed codons 19 to 104 and codons 489 to 694 of the *ICP0* gene. Thus, HSV-2 0ΔNLS encoded a GFP-tagged protein that lacked ICP0's NLS region and a portion of the C-terminal oligomerization domain (Fig. 1A, 1B). Finally, HSV-2 0Δ254 and HSV-2 0Δ810 contained deletions in the *ICP0* gene that resulted in expression of little more than GFP from the *ICP0* locus (Fig. 1A, 1B).

Southern blot analysis confirmed that each HSV-2 mutant virus bore an insertion of *GFP* coding sequence in place of a deletion of the expected size in the *ICP0* gene (Fig. 1A, 1C). Western blot analysis with a GFP-specific antibody confirmed that HSV-2 0Δ254 and HSV-2 0Δ810 encoded GFP-tagged, ICP0 peptides that were only slightly larger than native GFP expressed by an HSV-2 recombinant virus, HSV-2 MS-GFP (Fig. 1D, Fig. S2). As expected, HSV-2 0Δ104 and HSV-2 0ΔNLS encoded their respective 125 kDa and 85 kDa GFP-tagged proteins (Fig. 1D). In addition, 5 lower MW peptides of 45–80 kDa also reacted with GFP antibody (Fig. 1D). HSV-2 0ΔRING encoded an ∼120 kDa GFP-tagged protein, and only a single additional peptide was observed (Fig. 1D). ICP0 ubiquitinates itself in a RING-finger-dependent manner [44]; hence, most of the lower MW peptides observed in cells infected with HSV-2 0Δ104 or 0ΔNLS appeared to be the result of autoubiquitination and proteolytic turnover of GFP-tagged ICP0 (Fig. 1D). Collectively, Southern and Western blot analysis indicated that the intended mutations were successfully introduced into the HSV-2 *ICP0* gene.

### ICP0 is essential for HSV-2's resistance to interferon-α/β

**Figure 2:**
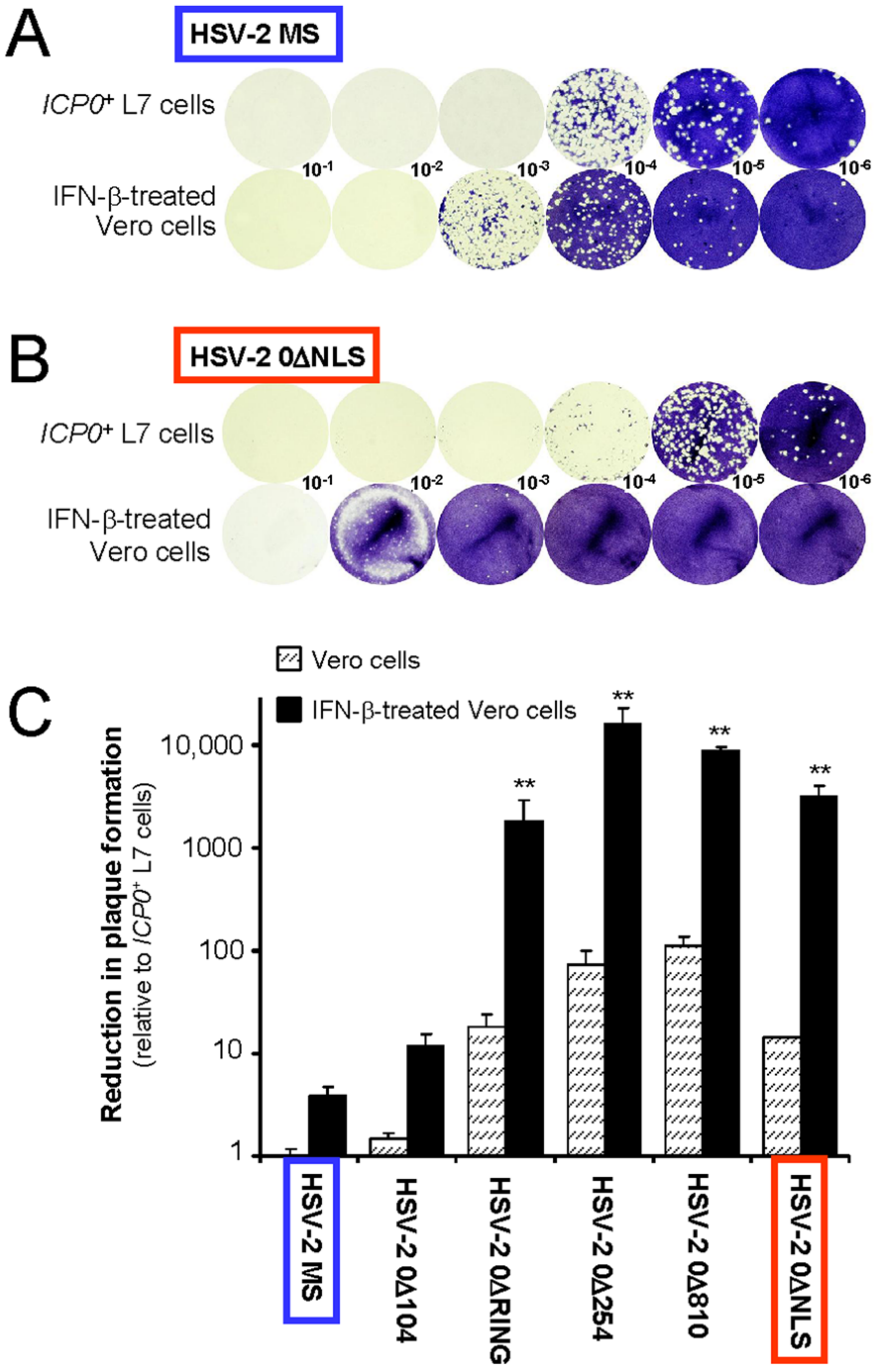
Plaque assay evaluation of interferon-sensitivity. (**A and B**) Photographs of monolayers of *ICP0* ^+^ L7 cells versus Vero cells treated with interferon-β (IFN-β, 200 U/ml), which were inoculated with 1.0-log dilutions of (A) wild-type HSV-2 MS or (B) HSV-2 0ΔNLS. (**C**) Reductions in the efficiency of plaque formation of each virus in untreated Vero cells or IFN-β-treated Vero cells relative to fully permissive monolayers of *ICP0* ^+^ L7 cells (mean ± sem reduction based on n = 3 plaque assays per group). A double asterisk (**) denotes p<0.001 regarding the probability, p, that each HSV-2 *ICP0* ^−^ mutant virus formed plaques in IFN-β-treated Vero cells with the same efficiency as wild-type HSV-2.

HSV-1 *ICP0* ^−^ null viruses do not replicate efficiently in cells that have been exposed to interferon (IFN)-α/β [36], [45]. To determine if HSV-2 *ICP0* ^−^ mutant viruses were also IFN-sensitive, wild-type HSV-2 MS and HSV-2 *ICP0* ^−^ mutant viruses were compared for their capacity to form plaques in monolayers of ICP0-complementing L7 cells, non-complementing Vero cells, or IFN-β-treated Vero cells. Wild-type HSV-2 MS (*ICP0* ^+^) formed plaques with equal efficiency in Vero and *ICP0* ^+^ L7 cells, and formed plaques 4-fold less efficiently in Vero cells treated with IFN-β (Fig. 2A, 2C). HSV-2 0Δ104 was only modestly attenuated, and formed plaques 12-fold less efficiently in Vero cells treated with IFN-β relative to *ICP0* ^+^ L7 cells (Fig. 2C). In contrast, all other HSV-2 *ICP0* ^−^ mutant viruses were acutely sensitive to IFN-α/β. For example, HSV-2 0ΔNLS formed plaques 14-fold less efficiently in Vero cells than *ICP0* ^+^ L7 cells, and formed plaques 3,300-fold less efficiently in Vero cells treated with IFN-β (Fig. 2B, 2C). Likewise, the HSV-2 *ICP0* ^−^ mutant viruses 0ΔRING, 0Δ254, and 0Δ810 formed plaques 2,000- to 15,000-fold less efficiently in IFN-β-treated Vero cells relative to *ICP0* ^+^ L7 cells (Fig. 2C).

**Figure 3:**
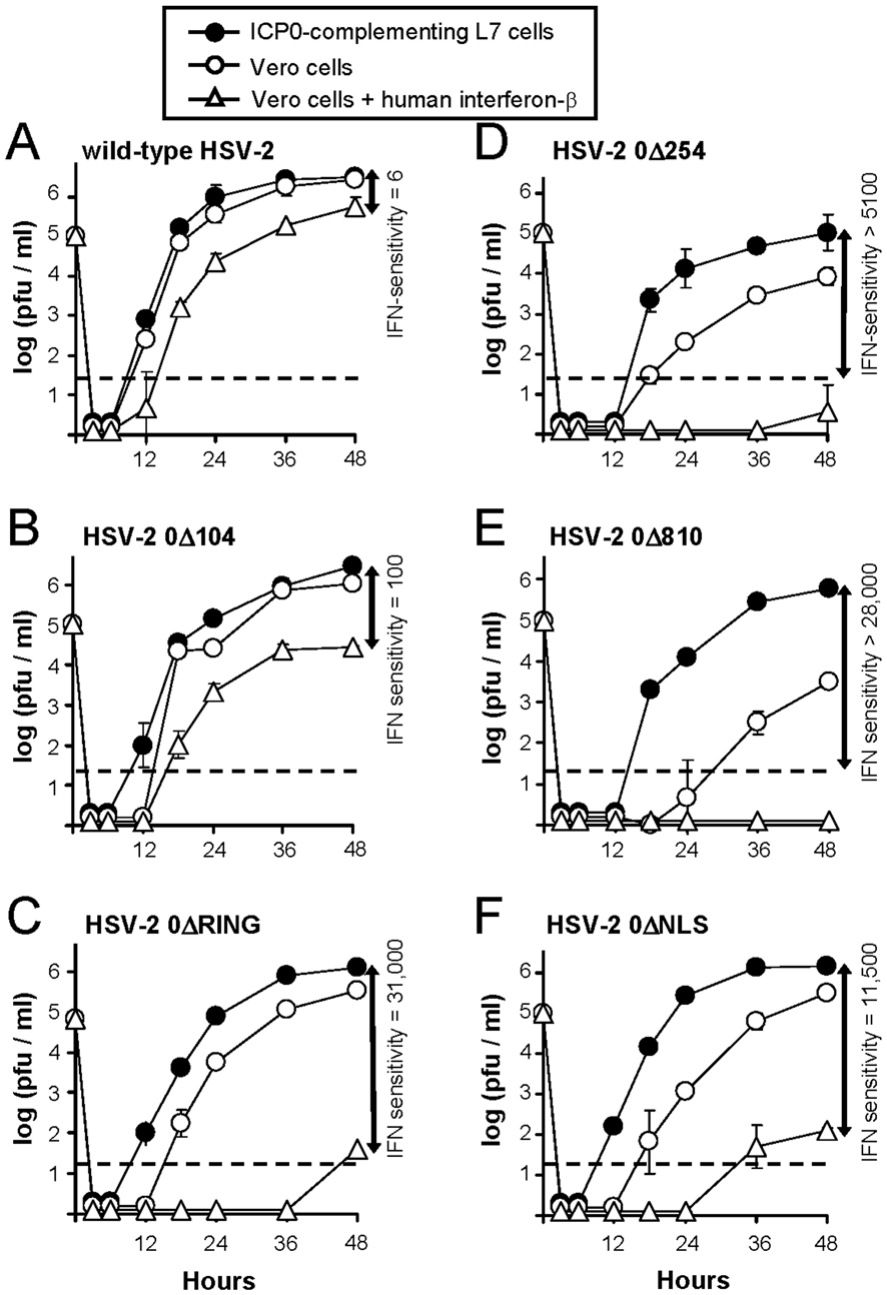
Replication efficiency of wild-type HSV-2 versus HSV-2 *ICP0* ^−^ mutant viruses. Titers of (**A**) wild-type HSV-2 MS, (**B**) HSV-2 0Δ104, (**C**) HSV-2 0ΔRING, (**D**) HSV-2 0Δ254, (**E**) HSV-2 0Δ810, or (**F**) HSV-2 0ΔNLS at times after inoculating *ICP0* ^+^ L7 cells, Vero cells, or interferon-β-treated Vero cells with 0.1 pfu per cell. At each indicated time point, cultures were transferred from 37°C to −80°C, and titers of infectious virus were determined upon thawing by a microtiter plaque assay. The mean ± sd of viral titer at each time point (n = 2 per data point) is plotted as a function of time. Arrows on the right of each graph represent the antilogarithm (10^x^) of the difference at 48 hours p.i. between viral titer in *ICP0* ^+^ L7 cells versus interferon-β-treated Vero cells. Each graph is a representative result from 2 to 3 independent experiments.

The growth kinetics of wild-type HSV-2 MS and HSV-2 *ICP0* ^−^ mutant viruses were compared in monolayers of *ICP0* ^+^ L7 cells, Vero cells, or IFN-β-treated Vero cells inoculated with 0.1 viral plaque-forming units (pfu) per cell. Wild-type HSV-2 grew to similar titers in L7 cells and Vero cells (Fig. 3A). The kinetics of HSV-2 MS replication were delayed in IFN-β-treated cells, but HSV-2 MS achieved a final titer that was only 6-fold lower than that observed in *ICP0* ^+^ L7 cells (Fig. 3A). HSV-2 0Δ104 was modestly attenuated, and grew to 100-fold lower titers in IFN-β-treated Vero cells relative to *ICP0* ^+^ L7 cells (Fig. 3B). In contrast, the other HSV-2 *ICP0* ^−^ mutant viruses 0ΔRING, 0Δ254, 0Δ810, and 0ΔNLS were acutely sensitive to IFN-α/β, and grew to 5,000- to 31,000-fold lower titers in IFN-β-treated Vero cells relative to *ICP0* ^+^ L7 cells (Fig. 3C–F). In the absence of IFN-β, HSV-2 0Δ254 and 0Δ810 replicated poorly in untreated Vero cells and achieved final titers of less than 10^4^ pfu/ml (Fig. 3D, 3E). In contrast, HSV-2 0Δ104, 0ΔRING, and 0ΔNLS each replicated to titers of greater than 10^5^ pfu per ml in untreated Vero cells (Fig. 3B, 3C, and 3F). Thus, HSV-2 0ΔRING and 0ΔNLS were the only HSV-2 *ICP0* ^−^ mutant viruses that were IFN-sensitive and retained the capacity to replicate with reasonable efficiency in cells that did not provide wild-type ICP0 *in trans*.

### HSV-2 0ΔNLS is severely attenuated relative to its ICP0^+^ parent virus, HSV-2 MS

**Figure 4:**
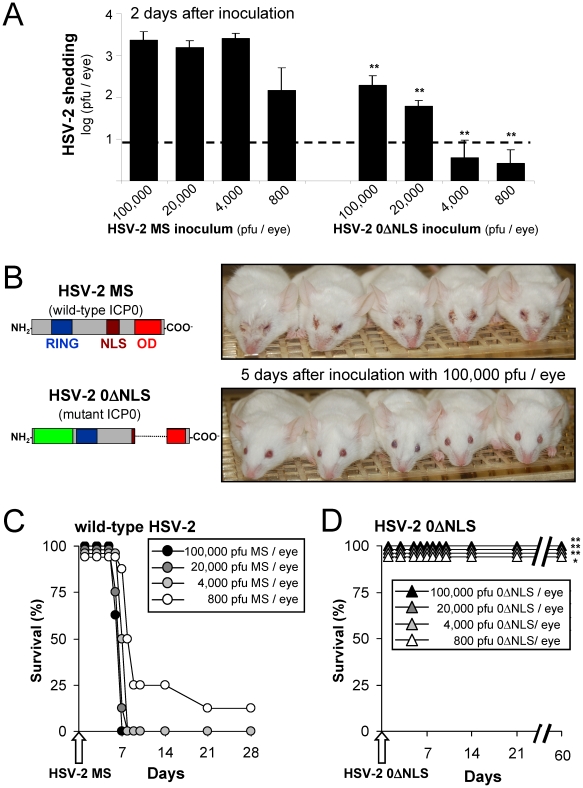
The 0ΔNLS mutation renders HSV-2 avirulent in outbred ICR mice. (**A**) HSV-2 shedding from the eyes of mice on Day 2 following bilateral ocular inoculation with 0.8, 4, 20, or 100 thousand pfu/eye of HSV-2 MS or HSV-2 0ΔNLS (n = 8 mice per group). (**B**) Representative photographs of naïve mice on Day 5 following ocular inoculation with 100,000 pfu/eye of wild-type HSV-2 MS versus the HSV-2 *ICP0* ^−^ mutant virus, HSV-2 0ΔNLS. (**C and D**) Duration of survival of naïve mice following ocular inoculation with 0.8, 4, 20, or 100 thousand pfu/eye of (B) HSV-2 MS or (C) HSV-2 0ΔNLS (n = 8 mice per virus per dose; Σn = 32 mice per virus). A single asterisk (*) denotes p<0.05, and a double asterisk (**) denotes p<0.001 regarding the probability, p, that HSV-2 shedding or survival was equivalent in groups of mice inoculated with the same dose of HSV-2 MS or HSV-2 0ΔNLS.

A dose of 500 or more pfu of wild-type HSV-1 readily establishes productive infections in the eyes of mice, whereas 20-fold higher doses of HSV-1 *ICP0* ^−^ mutant viruses are required to establish a productive infection in mouse eyes [46]. To determine the relative doses of wild-type HSV-2 or HSV-2 *ICP0* ^−^ mutant virus required to establish a productive infection in mouse eyes, outbred ICR mice were inoculated bilaterally with 0.8, 4, 20, or 100 thousand pfu per eye of wild-type HSV-2 MS strain or HSV-2 0ΔNLS (n = 8 per virus per dose). At all viral doses tested, wild-type HSV-2 MS established robust infections that led to HSV-2 shedding in mouse eyes by Day 2 post-inoculation (p.i.) and produced overt periocular disease by Day 5 p.i. (Fig. 4A, 4B). Wild-type HSV-2 MS infections were lethal in 31 of 32 ICR mice (Fig. 4C). These results were consistent with independent tests in which the 50% lethal dose (LD_50_) of HSV-2 MS was found to be ∼200 pfu per eye (not shown).

HSV-2 0ΔNLS required higher doses of virus to establish a productive infection in outbred ICR mice. At doses of 800 or 4,000 pfu per eye, less than 50% of mice inoculated with HSV-2 0ΔNLS shed virus on Day 2 p.i. (Fig. 4A). However, at doses of 20,000 or 100,000 pfu per eye, ocular shedding of HSV-2 0ΔNLS was observed in all mice on Day 2 p.i. (Fig. 4A). Mice inoculated with HSV-2 0ΔNLS remained indistinguishable from uninfected mice between Days 5 and 60 p.i. (Fig. 4B, 4D). Therefore, the LD_50_ of HSV-2 0ΔNLS was at least 500 times greater than HSV-2 MS in an eye model of infection. Based on these results, a dose of 100,000 pfu per eye was used to inoculate mice with HSV-2 *ICP0* ^−^ mutant viruses in subsequent tests.

### Interferon-sensitive, HSV-2 ICP0^−^ mutant viruses establish inapparent infections

**Figure 5:**
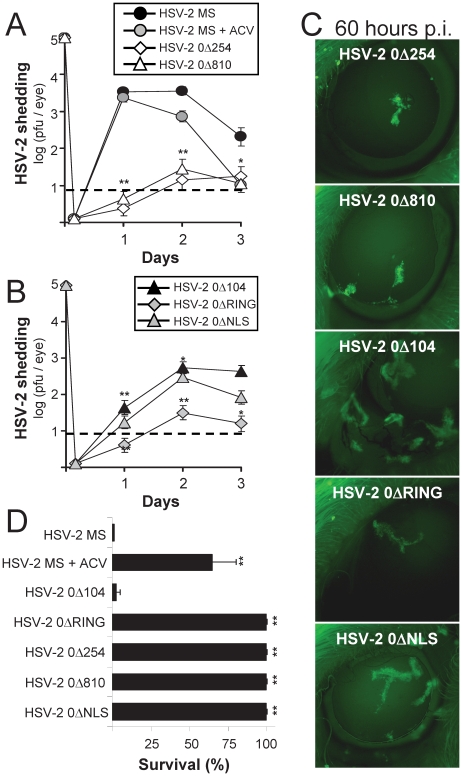
Interferon-sensitive HSV-2 *ICP0* ^−^ mutant viruses produce inapparent infections in mice. (**A**) HSV-2 shedding from the right eyes of mice inoculated with 100,000 pfu/eye of wild-type HSV-2 MS which were untreated or were treated with the antiviral drug acyclovir (ACV), as compared to mice inoculated in the right eye with (**A**) HSV-2 0Δ254 or HSV-2 0Δ810 or (**B**) HSV-2 0Δ104, HSV-2 0ΔNLS, or HSV-2 0ΔRING (n = 15 per group). ACV-treated mice were ***i.*** intraperitoneally administered 30 mg/kg ACV on Days −1, 0, 1, and 3 p.i., and were ***ii.*** orally administered 1 mg/ml ACV in their drinking water from Days −3 to +20 p.i. A double asterisk (**) denotes p<0.001 regarding the probability, p, that viral shedding on a given day was equivalent to HSV-2 MS-infected mice that received no ACV. (**C**) Representative sites of GFP-tagged ICP0 mutant protein expression in mouse corneas inoculated with HSV-2 *ICP0* ^−^ mutant viruses at 60 hours p.i. (**D**) Mean ± sd frequency of survival following inoculation with wild-type HSV-2 MS or HSV-2 *ICP0* ^−^ mutant viruses in two independent experiments, as determined on Day 30 p.i. (Σn = 25 mice per group). A double asterisk (**) denotes p<0.001 regarding the probability, p, that the frequency of survival was equivalent to HSV-2 MS-infected mice that received no ACV.

The efficiency of replication of HSV-2 *ICP0* ^−^ mutant viruses was compared to wild-type HSV-2 following inoculation of the right eye with a viral dose of 100,000 pfu per eye (Fig. 5). To ensure that a subset of mice survived infection with wild-type HSV-2, one group of HSV-2 MS-infected mice was aggressively treated with acyclovir (ACV) both orally and intraperitoneally between Days −3 and +20 p.i. to limit viral spread and pathogenesis [47], [48], [49]. All mice inoculated with HSV-2 MS (*ICP0* ^+^) shed more than 3,000 pfu from the right eye on Day 1 p.i., and ACV treatment only modestly reduced HSV-2 shedding on Days 2 and 3 p.i. (Fig. 5A). Each of the five mutations in HSV-2's *ICP0* gene resulted in a significant reduction of HSV-2 shedding between Days 1 and 3 p.i. (Fig. 5A, 5B). Specifically, mice inoculated with HSV-2 0Δ254, 0Δ810, or 0ΔRING shed less than 100 pfu per eye between Days 1 and 3 p.i. (Fig. 5A, 5B). Likewise, mice inoculated in the right eye with HSV-2 0Δ104 or HSV-2 0ΔNLS shed peak titers of ∼300 pfu per eye on Day 2 p.i. (Fig. 5B).

Expression of GFP-tagged, mutant ICP0 was visualized in corneas at 60 hours p.i. by fluorescent microscopy (Fig. 5C). All corneas inoculated with HSV-2 *ICP0* ^−^ mutant viruses exhibited at least small foci of cells expressing GFP-tagged, mutant ICP0 (Fig. 5C; n = 25 eyes observed per group). However, corneas inoculated with HSV-2 0Δ104 or HSV-2 0ΔNLS contained wider tracts of GFP^+^ cells that were consistently >10 cells across (Fig. 5C).

The frequency of survival of HSV-2 infected mice was compared on Day 30 p.i. In untreated mice, ocular inoculation with HSV-2 MS was uniformly lethal by Day 8 p.i. (Fig. 5D). Aggressive use of ACV reduced mortality, and hence 21 of 30 ACV-treated mice survived HSV-2 MS infection until Day 30 p.i. (Fig. 5D). HSV-2 0Δ104 was modestly attenuated, and thus mice inoculated with HSV-2 0Δ104 survived 4 days longer than mice inoculated with wild-type HSV-2 MS (i.e., duration of survival = 11±1 days p.i.). Nonetheless, only 1 of 25 mice survived ocular inoculation HSV-2 0Δ104 (Fig. 5D). In contrast, 25 of 25 mice survived inoculation of the right eye with HSV-2 0ΔRING, 0Δ254, 0Δ810, or 0ΔNLS, and these mice remained disease-free between Days 5 and 60 p.i. (Fig. 5D). Therefore, all HSV-2 *ICP0* ^−^ mutant viruses that were IFN-sensitive in cell culture (Fig. 2C) were also avirulent in mice (Fig. 5D).

### Mice inoculated with HSV-2 0ΔNLS are consistently protected against wild-type HSV-2

**Figure 6:**
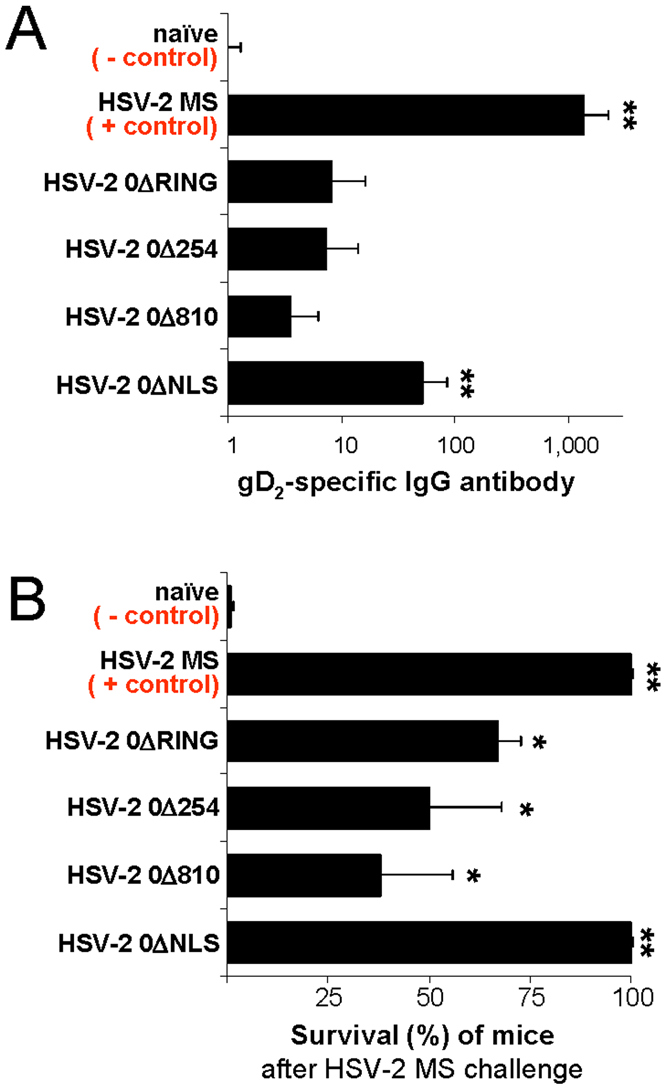
Inoculation with HSV-2 0ΔNLS imparts protective immunity against HSV-2. (**A**) Mean ± sem titer of serum IgG antibody specific for HSV-2 glycoprotein D (gD_2_), as determined by ELISA on 1∶100 dilutions of mouse serum (n = 20 per group). (**B**) Mean ± sd of the frequency of survival of mice until 30 days after challenge of the left eye with a dose of 100,000 pfu/eye of HSV-2 MS (n = 8 mice per group per test×2 tests; Σn = 16 per group). A single asterisk (*) denotes p<0.05, and a double asterisk (**) denotes p<0.001 regarding the probability, p, that the survival frequency of each group was equivalent to naïve mice.

HSV-2-specific IgG levels and protective immunity were compared among the 100% of mice that survived inoculation with HSV-2 *ICP0* ^−^ mutant viruses and the 70% of ACV-treated mice that survived primary infection with wild-type HSV-2 MS (Fig. 5D). Mice were bled on Day 60 p.i. and sera were tested for the presence of IgG antibody against recombinant HSV-2 glycoprotein D (gD_2_) [50]. Dilutions of pooled HSV-2 antiserum defined the quantitative relationship between color development and gD_2_-antibody abundance (r^2^ = 1.00; Fig. S3). The serum of naïve mice defined the background of the assay, and HSV-2 MS latently infected mice served as a positive control group that should possess *bona fide* immunity to secondary HSV-2 infection (Fig. 6A). HSV-2 MS latently infected mice possessed levels of gD_2_-antibody that were ∼1,350-fold above background (Fig. 6A, p<0.001). Only ∼50% of mice inoculated with HSV-2 0ΔRING, 0Δ254, or 0Δ810 possessed detectable gD_2_-antibody, and thus their gD_2_-antibody responses did not significantly differ from background (Fig. 6A; p>0.05). In contrast, the level of gD_2_-antibody in HSV-2 0ΔNLS-inoculated mice was an average 53-fold above background, and thus represented 4% of that observed in HSV-2 MS latently infected mice (Fig. 6A; p<0.001).

On Days 70 and 80 after inoculating *the right eye* with HSV-2 *ICP0* ^−^ mutant viruses, mice were secondarily challenged *in the left eye* with 500 times the LD_50_ of HSV-2 MS (i.e., 100,000 pfu per eye). The summated results of these two challenge experiments are presented (Σn = 16 mice per group). As expected, 0±0% of naïve mice survived for more than 8 days post-challenge (Fig. 6B). Consistent with their robust gD_2_-antibody responses, 100±0% of HSV-2 MS latently infected mice survived superinfection of the left eye with HSV-2 MS, and did not exhibit any symptoms of disease following challenge (Fig. 6B). Mice inoculated with HSV-2 0ΔRING, 0Δ254, or 0Δ810 were not consistently protected against wild-type HSV-2 MS challenge, and their survival rates were 70±10%, 50±20%, and 40±20%, respectively (Fig. 6B). In contrast, 100±0% of mice inoculated with HSV-2 0ΔNLS survived lethal challenge with HSV-2 MS, and 11 of 16 of these mice survived disease-free for 30 days post-challenge (Fig. 6B). Therefore, HSV-2 0ΔNLS was the only virus tested that consistently ***1.*** replicated at the site of inoculation, ***2.*** established an inapparent infection in mice, and ***3.*** elicited robust protection in mice against later exposures to wild-type HSV-2.

### The attenuated *in vivo* phenotype of HSV-2 ICP0^−^ mutant viruses is Stat 1-dependent

**Figure 7:**
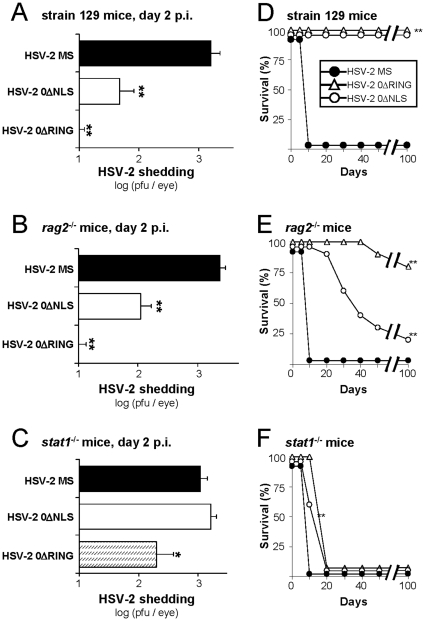
HSV-2 *ICP0* ^−^ mutant viruses are repressed by a Stat 1-dependent host response. (**A–C**) Viral shedding on Day 2 p.i. from the eyes of (A) wild-type strain 129 mice, (B) lymphocyte-deficient *rag2* ^−/−^ mice, or (C) IFN-signaling-deficient *stat1* ^−/−^ mice inoculated bilaterally with 100,000 pfu/eye of wild-type HSV-2 MS, HSV-2 0ΔNLS, or HSV-2 0ΔRING (n = 10 mice per group). A single asterisk (*) denotes p<0.05 and a double asterisk (**) denotes p<0.001 regarding the probability, p, that viral shedding was equivalent to HSV-2 MS shedding in the same strain of mice. (**D–F**) Duration of survival of (D) strain 129 mice, (E) *rag2* ^−/−^ mice, or (F) *stat1* ^−/−^ mice following inoculation with wild-type HSV-2 MS, HSV-2 0ΔNLS, or HSV-2 0ΔRING (n = 10 mice per group). A double asterisk (**) denotes p<0.001 regarding the probability, p, that the duration of survival was equivalent to HSV-2 MS-infected mice of the same mouse strain.

HSV-2 0ΔNLS and HSV-2 0ΔRING were IFN-sensitive in cultured cells and avirulent in mice (Fig. 2, 3, 5). To determine if these phenotypes were causally related, viral replication and disease were compared in ***i.*** wild-type strain 129 mice, ***ii.*** lymphocyte-deficient *rag2* ^−/−^ mice, or ***iii.*** IFN-signaling-deficient *stat1* ^−/−^ mice. Regarding the latter group, absence of the signal transducer and activator of transcription 1 (Stat 1) results in a signaling defect in which ligand binding to IFN receptors fails to induce the transcription of the >300 IFN-stimulated genes that mediate the ‘antiviral state’ [51], [52], [53]. Mice were inoculated bilaterally with 100,000 pfu/eye of HSV-2 MS, 0ΔNLS, or 0ΔRING, and HSV-2 shedding was compared across all groups of mice on Day 2 p.i. (Fig. 7A–C). Wild-type strain 129 mice shed ∼1,600 pfu/eye of wild-type HSV-2 MS. In contrast, strain 129 mice inoculated with HSV-2 0ΔNLS or HSV-2 0ΔRING shed ∼50 and 7 pfu/eye, respectively (Fig. 7A). Lymphocyte-deficient *rag2* ^−/−^ knockout mice inoculated with these viruses exhibited an equivalent pattern of HSV-2 shedding on Day 2 p.i. (Fig. 7B). Hence, the restricted pattern of HSV-2 0ΔNLS and 0ΔRING shedding from mouse eyes was not dependent on host lymphocytes (Fig. 7A vs 7B). In contrast, *stat1* ^−/−^ mice shed ∼1,500 pfu/eye of both wild-type HSV-2 MS and HSV-2 0ΔNLS on Day 2 p.i. (Fig. 7C; p>0.05). Likewise, *stat1* ^−/−^ mice shed ∼200 pfu/eye of HSV-2 0ΔRING, which represented a 30-fold increase in HSV-2 0ΔRING shedding relative to strain 129 mice (Fig. 7A vs 7C). Thus, an innate, Stat 1-dependent host response severely restricted HSV-2 0ΔNLS and 0ΔRING shedding in strain 129 mice and *rag2* ^−/−^ mice on Day 2 p.i..

Disease and survival were compared among mice inoculated with HSV-2 MS, 0ΔNLS, or 0ΔRING. As expected, HSV-2 MS produced fatal encephalitis in all mice within 8 days p.i. (Fig. 7D–F). HSV-2 0ΔNLS and 0ΔRING established inapparent infections in 100% of strain 129 mice (Fig. 7D). HSV-2 0ΔNLS and 0ΔRING produced infections that slowly progressed towards lethal disease in *rag2* ^−/−^ mice (Fig. 7E). HSV-2 0ΔNLS infection of *rag2* ^−/−^ mice progressed to a fatal condition in 80% of *rag2* ^−/−^ mice between Days 18 and 50 p.i. (Fig. 7E). In contrast, HSV-2 0ΔRING infection was fatal in only 2 of 10 *rag2* ^−/−^ mice over a 100-day observation period (Fig. 7E). Presumably, the slower rate at which HSV-2 0ΔRING infection caused disease in *rag2* ^−/−^ mice reflected a more severe defect in the mutant ICP0^ΔRING^ protein, as ICP0's RING finger domain is essential for all of ICP0's known activator functions [32], [33], [34]. In contrast, loss of IFN-signaling in *stat1* ^−/−^ mice was associated with more rapid disease progression. *Stat1* ^−/−^ mice succumbed to HSV-2 0ΔNLS or 0ΔRING infection between Days 9 and 18 p.i. (Fig. 7F). Thus, the attenuated phenotype of HSV-2 *ICP0* ^−^ mutant viruses was only observed in animals that possessed the capacity to mount an innate, Stat 1-dependent host antiviral response [35], [37], [54].

### HSV-2 0ΔNLS infections fail to spread from the site of inoculation

We were interested in clarifying how HSV-2 0ΔNLS consistently replicated in mouse eyes without producing disease. We considered the possibility that the 0ΔNLS mutation might reduce the spread of HSV-2 0ΔNLS infection. To test this hypothesis, we compared the spread of HSV-2 0ΔNLS (*ICP0* ^−^) infection across mouse faces relative to an *ICP0* ^+^ virus that also expressed a fluorescent marker, HSV-2 MS-GFP (Fig. S2).

**Figure 8:**
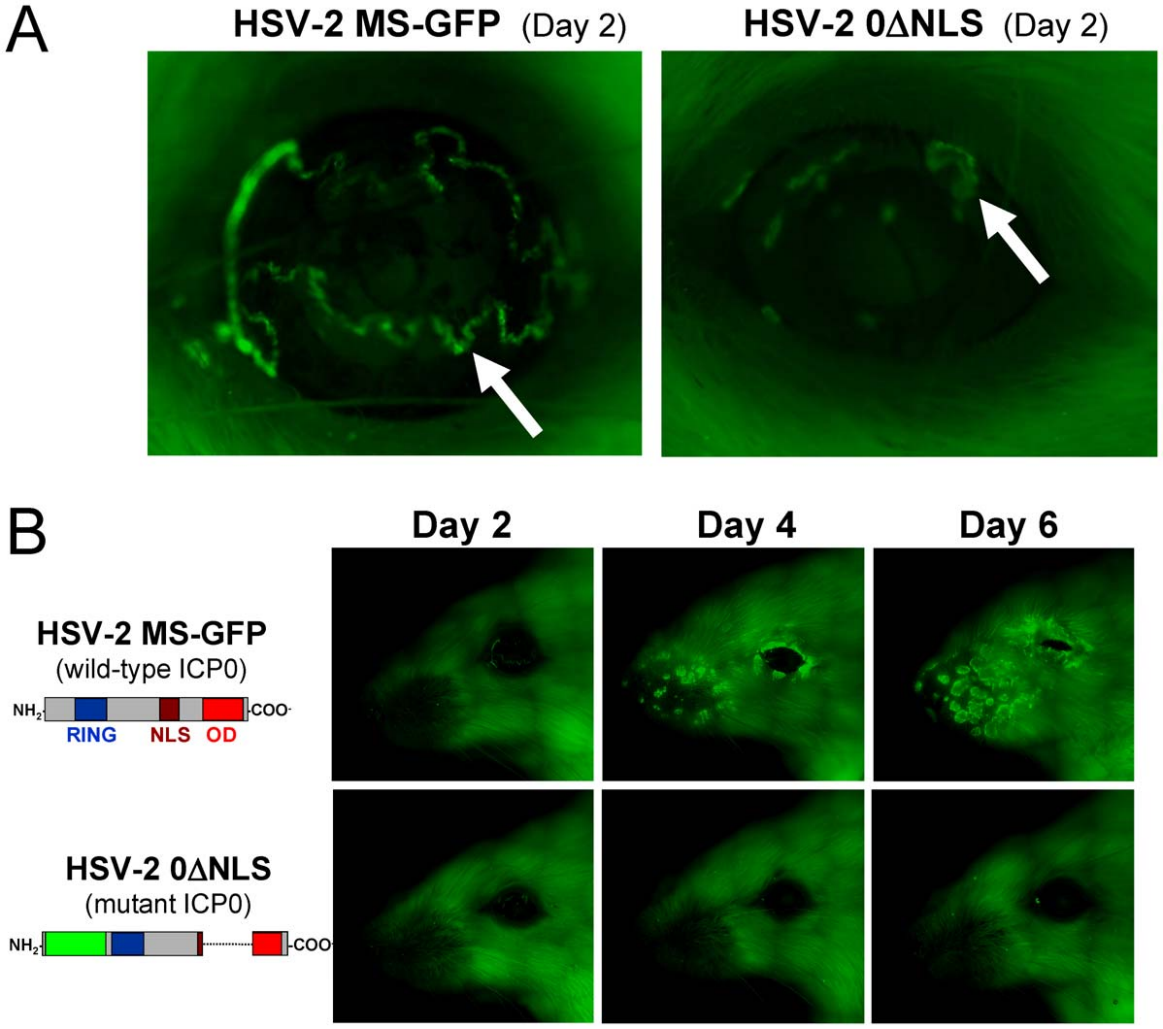
HSV-2 0ΔNLS replicates at the site of inoculation, but fails to spread. (**A**) Sites of GFP expression (arrows), as visualized on Day 2 p.i., in the eyes of ICR mice inoculated with 100,000 pfu/eye of HSV-2 MS-GFP or HSV-2 0ΔNLS. (**B**) Progression of the spread of GFP expression across the faces of mice inoculated with HSV-2 MS-GFP or HSV-2 0ΔNLS as visualized on Days 2, 4, and 6 p.i. These experiments were performed on n = 3 mice per group, and the progression of infection is shown in a single representative mouse per group.

On Day 2 after bilateral inoculation with 100,000 pfu per eye of HSV-2 MS-GFP, GFP expression was only observed at the site of inoculation (Fig. 8A, 8B; Fig. S4). On day 4 p.i., circular foci of HSV-2 MS replication (GFP^+^ cells) were evident on the noses of mice, and these circular lesions enlarged and merged by Day 6 p.i. (Fig. 8B). This rapid spread of HSV-2 MS-GFP infection was accompanied by intense inflammation of the epithelium, and was rapidly followed by fatal encephalitis.

On Day 2 after bilateral inoculation with 100,000 pfu/eye of HSV-2 0ΔNLS, GFP expression was only observed at the site of inoculation (Fig. 8A, 8B; Fig. S4). HSV-2 0ΔNLS did not visibly spread from the site of inoculation, and thus GFP^+^ cells were not observed in the eyes of mice or in the periocular epithelium on Days 4, 6, or 8 p.i. (Fig. 8B, Fig. S4; Day 8 not shown). Thus, HSV-2 0ΔNLS replicated briefly at the site of inoculation, but failed to spread or cause visible inflammation and disease.

## Discussion

### HSV-2 ICP0^−^ mutant viruses that are interferon-sensitive are severely attenuated *in vivo*

Prior studies have established that HSV-1 *ICP0* ^−^ mutant viruses are interferon-sensitive, avirulent, and elicit protective immunity against HSV-1 [37]. Based on these observations, we have inferred that HSV-2 *ICP0* ^−^ mutant viruses might be useful as a live-attenuated vaccine to prevent genital herpes [13], [37]. Understandably, enthusiasm for this proposal has been limited by the absence of data describing HSV-2 *ICP0* ^−^ mutant viruses. The results of the current study address this gap in knowledge.

HSV-2 MS infection of the eye is almost invariably lethal in naïve ICR mice. In contrast, recombinant HSV-2 *ICP0* ^−^ mutant viruses that were IFN-sensitive (Fig. 2, 3) were also avirulent in ICR mice (Fig. 4, 5). Hence, mutations in the *ICP0* gene are sufficient to attenuate even a highly virulent HSV-2 strain such as MS. One HSV-2 MS-derived *ICP0* ^−^ mutant, HSV-2 0Δ104, was not attenuated and produced lethal disease in 24 of 25 mice (Fig. 5D). In hindsight, this outcome was predictable based on HSV-2 0Δ104's resistance to IFN-inducible repression in cultured cells (Fig. 2, 3). The correlation between IFN-sensitivity and avirulence is unlikely to be a coincidence. HSV-1 *ICP0* ^−^ mutant viruses are avirulent in immunocompetent mice, but acquire the ability to replicate efficiently and cause disease when host IFN signaling pathways are compromised [35], [37]. Based on this precedent, we were able to correctly predict that the avirulent phenotype of HSV-2 0ΔNLS would be dependent on the IFN-signal transducing factor, Stat 1 (Fig. 7). Thus consistent with the established properties of HSV-1 *ICP0* ^−^ viruses, the IFN-induced antiviral state appears to be essential to the avirulent phenotype of IFN-sensitive HSV-2 *ICP0* ^−^ mutant viruses in immunocompetent animals.

### Are the phenotypes of mutant HSV-2 viruses a consequence of mutations in the ICP0 gene?

Four lines of evidence indicate that the phenotypes of HSV-2 0ΔNLS are a consequence of the intended mutation in the *ICP0* gene, and this data is considered, as follows. First, Southern blot and Western blot analysis verify that the predicted genetic change was introduced into the *ICP0* locus of HSV-2 0Δ104, 0ΔRING, 0ΔNLS, 0Δ254, and 0Δ810 (Fig. 1). Second, the phenotypes of IFN-sensitivity and avirulence are shared by four independent HSV-2 *ICP0* ^−^ mutant viruses; HSV-2 0ΔRING, 0ΔNLS, 0Δ254, and 0Δ810. It is improbable that four independent HSV-2 *ICP0* ^−^ mutant viruses would each acquire the expected phenotypes of IFN-sensitivity and avirulence based on secondary mutations randomly scattered throughout portions of the HSV-2 genome outside of the *ICP0* locus. Third, it was previously predicted that HSV-2 *ICP0* ^−^ mutant viruses should be IFN-sensitive and avirulent [13], [37] based on the fact that multiple HSV-1 *ICP0* ^−^ mutant viruses exhibit these phenotypes [35], [36], [45]. Finally, whatever genetic defect impairs the replication of HSV-2 0ΔRING, 0ΔNLS, 0Δ254, and 0Δ810 in Vero cells is functionally complemented when wild-type ICP0 is provided *in trans* from the *ICP0* ^+^ Vero-derived cell line, L7 cells (Fig. 3). Therefore, mutations within the *ICP0* gene of HSV-2 0ΔNLS appear to be responsible for the observed phenotypes of IFN-sensitivity and avirulence.

### HSV-2 ICP0^−^ mutant viruses that lack ICP0's RING finger region are overattenuated

HSV-2 *ICP0* ^−^ mutant viruses that included deletions of ICP0's RING finger domain were avirulent, but did not elicit consistent protection against HSV-2 (Fig. 6B). Thus, deletions in ICP0's RING finger region produced HSV-2 viruses that appeared to be overattenuated, and not particularly likely to succeed as a live-attenuated HSV-2 vaccine strain. In contrast, HSV-2 0ΔNLS exhibited the properties of a desirable live-attenuated HSV-2 vaccine strain. HSV-2 0ΔNLS consistently replicated at sites of ocular inoculation, and was avirulent in immunocompetent mice. Due to its consistent replication, HSV-2 0ΔNLS consistently stimulated an immune response in recipient mice, as measured by IgG antibodies against HSV-2's immunodominant gD antigen, and protective immunity against wild-type HSV-2 (Fig. 6).

The results suggest that HSV-2 replication at the site of immunization may be proportional to the magnitude of the resulting immune response. For example, mice inoculated with HSV-2 0ΔRING shed peak titers of virus that were ∼1% of the peak titers shed by HSV-2 MS-infected mice and mounted gD_2_-antibody responses that were ∼0.5% of that in HSV-2 MS-infected mice (Fig. 5, 6). Likewise, mice inoculated with HSV-2 0ΔNLS shed peak titers of virus that were 10-fold higher than the peak titer of virus shed by HSV-2 0ΔRING-infected mice, and mounted a gD_2_-antibody response that was ∼10-fold greater than HSV-2 0ΔRING and which was 1/25^th^ of that elicited by HSV-2 MS (Fig. 5, 6). Therefore, the average peak titer of virus shed following inoculation (Fig. 5A, 5B) was roughly predictive of the average gD_2_-antibody response observed on Day 60 p.i. (Fig. 6A). Importantly, serum levels of gD_2_-antibody correlated with functional protection against HSV-2 MS challenge (Fig. 6A vs 6B).

### Correlation between gD_2_-antibody titers and protective immunity against HSV-2?

When HSV-2 gD has been used as a subunit vaccine in human clinical trials, the potency of the resulting gD_2_-antibody response does not correlate with protective immunity against HSV-2 [55], [56], [57]. This observation appears to contradict our finding that mice immunized with live HSV-2 viruses generate a protective immune response that varies in proportion to serum levels of gD_2_-antibody (Fig. 6A, 6B). Two explanations may resolve this discrepancy. First, *mouse* IgG antibodies against HSV-2 may be more protective than *human* IgG antibodies against HSV-2 [58]. Thus, the correlation between gD_2_-antibody titers and protection against HSV-2 challenge may be an artefact of the mouse model used in this study. A second possibility is that HSV-2 gD subunit vaccines may be only weakly protective against HSV-2 infections regardless of the magnitude of the antibody response elicited against the gD_2_ subunit. We favor the latter explanation for two reasons.

First, immunization with an HSV-2 gD subunit vaccine is the equivalent of vaccinating an animal with ∼1% of HSV-2 (i.e., 1 of 80 HSV-2 proteins). In contrast, immunization with a live virus such as HSV-2 0ΔNLS may drive the clonal expansion of B and T lymphocytes specific for nearly the entire proteome of HSV-2, which consists of thousands of combinations of 6 to 10 amino-acid epitopes distributed across HSV-2's 80 proteins. The *increased breadth* of the immune response against a live-attenuated virus may explain why this mode of vaccination has been so effective in preventing severe viral diseases such as smallpox and measles.

Second, gD_2_-antibody responses provide a robust index of protective immunity against HSV-2 when mice are immunized with a live HSV-2 virus, but this is not the case when mice are immunized with an HSV-2 gD subunit vaccine (unpublished data of W. Halford). For example, mice immunized with an HSV-2 gD subunit vaccine mount a gD_2_-antibody response that is 20-fold greater than that elicited by immunization of mice with HSV-2 0ΔNLS (unpublished data of W. Halford). However, only 3 of 45 gD-immunized mice survive ocular or vaginal challenge with wild-type HSV-2, whereas 119 of 120 0ΔNLS-immunized mice survive ocular or vaginal challenge with wild-type HSV-2 (unpublished data of W. Halford). Based on our experience in mice, we infer that when a live HSV-2 virus is used as the immunogen, then serum levels of gD_2_-antibody provide a reliable index of protective immunity against HSV-2. It remains to be determined if this inference holds true in other species.

### HSV-2 latently infected mice as a positive control for vaccine-challenge studies

Most HSV-2 vaccine-challenge study designs only compare immunity to HSV-2 in vaccinated animals versus naïve controls [16], [18], [59], [60], [61]. Such study designs establish that an HSV-2 vaccine candidate elicits more than “0% immunity” against HSV-2. However, the efficacy of HSV-2 vaccine candidates might be more clearly defined on a scale of 0 to 100% immunity against HSV-2. Given that animals latently infected with wild-type HSV are resistant to superinfection [62], [63], [64], the efficacy of HSV-2 vaccine candidates could be described in terms of “% immunity” relative to such a positive control. In the current study, we describe the efficacy of the HSV-2 0ΔNLS vaccine candidate in such terms (Fig. 6A, 6B). Specifically, a single exposure to HSV-2 0ΔNLS elicited a gD_2_-antibody response that was 4% of that observed in wild-type HSV-2 MS latently infected mice (Fig. 6A). Likewise, HSV-2 0ΔNLS showed promise as a vaccine candidate because recipient mice survived lethal HSV-2 MS challenge at the same 100% frequency as HSV-2 MS latently infected mice (Fig. 6B). We propose that future vaccine studies might benefit from the inclusion of such a positive control that helps define the “100% target value” of a *bona fide* protective immune response against HSV-2.

### Safety concerns surrounding a vaccine based on a live-attenuated α-herpesvirus?

Many questions remain to be addressed about a live-attenuated HSV-2 vaccine strain. Is it possible that a live-attenuated virus such as HSV-2 0ΔNLS may recombine with endogenous or superinfecting HSV-2 viruses? Would HSV-2 0ΔNLS establish latent infections in vaccine recipients, or reactivate from the latent state? These are important questions that merit further study. However, an initial risk-benefit analysis may be performed in light of clinical experience with the live VZV vaccine. Like HSV-2, VZV is an α-herpesvirus that establishes life-long infections in human neurons. Due to the extensive similarity between these viruses, the safety concerns surrounding a live HSV-2 vaccine strain are similar to the Oka vaccine strain of VZV.

In the >20-year history of vaccinating against chickenpox, recombination between the VZV Oka strain and wild-type VZV has not been reported as a clinical problem. Rather, the major problem is that the VZV Oka strain may produce disease in recipients that are severely immunocompromised [27]. While the VZV Oka strain may establish latent infections in human neurons and reactivate from the latent state [28], clinical experience suggests that the risks associated with this live VZV vaccine are outweighed by the benefits of not leaving a population susceptible to the >90% risk of being infected with wild-type VZV [65]. If clinical experience with the VZV Oka strain is any indication, then the risks associated with a live-attenuated HSV-2 vaccine strain would likely be preferable to the current situation in which wild-type HSV-2 is carried by ∼1 billion people, and ∼20 million people are newly infected with these disease-causing strains of HSV-2 each year.

### Conclusion

Most vaccines that have succeeded in preventing viral disease have been based upon live, replicating viruses. Millions of children receive vaccines every year that contain live-attenuated variants of poliovirus, mumps virus, measles virus, rubella virus, and VZV. Despite these successes, efforts to develop a genital herpes vaccine have primarily focused on non-replicating HSV-2 vaccine candidates such as gD_2_-protein subunits [15], [16], [66], [67], [68] and replication-defective HSV-2 viruses [17], [18], [61], [69]. These approaches are extraordinarily safe, but have not slowed the spread of genital herpes. Perhaps it is time to consider the possibility that a live-attenuated HSV-2 vaccine strain might be more effective.

Deletion of conserved regions of ICP0 is a feasible approach to obtain a new class of live-attenuated HSV-2 vaccine strains that is safe in theory and practice due to their exquisite sensitivity to repression by the innate IFN system of the animal host. Therefore, we conclude that HSV-2 *ICP0* ^−^ mutant viruses merit further consideration for their potential to prevent the spread of HSV-2 and genital herpes in the human population.

## Materials and Methods

### Ethics Statement

Mice were handled in accordance with the National Institutes of Health Guide for the Care and Use of Laboratory Animals. This study was approved by the Southern Illinois University School of Medicine Laboratory Animal Care and Use Committee in August 2008, and was assigned Protocol Numbers #205-08-018 and #205-08-019. These protocols remain active and are associated with an NIH-funded grant for the “Development of an Effective Genital Herpes Vaccine (R21 AI081072).

### Cells and viruses

Vero cells and U2OS cells were obtained from the American Type Culture Collection (Manassas, VA), and High Five™ insect cells were obtained from Invitrogen Corporation (Carlsbad, CA). The ICP0-complementing L7 cell line [70] was kindly provided by Neal Deluca (University of Pittsburgh). Cell lines were propagated in Dulbecco's Modified Eagle's medium supplemented with 5% fetal bovine serum and antibiotics. The HSV-2 recombinant viruses used in this study were derivative of HSV-2 MS (American Type Culture Collection). Most HSV-2 lab strains and HSV-2 clinical isolates grow to ∼100-fold lower titers than HSV-1. To circumvent this problem, all HSV-2 viruses were propagated in U2OS cells at 34°C following inoculation with a multiplicity of infection of 0.01 pfu per cell, which generally resulted in an ∼10-fold increase in HSV-2 titers. For both wild-type HSV-2 and HSV-2 *ICP0* ^−^ mutant viruses, viral stocks were generated for animal experiments that were concentrated 10-fold by ultracentrifugation to achieve a minimum titer of 3×10^7^ pfu/ml. An HSV-2 glycoprotein D-expressing baculovirus was used to purify the gD-2 306t protein [50], and was generously provided by Dr. Gary Cohen and Dr. Roslyn Eisenberg (University of Pennsylvania).

### Plasmid precursors of HSV-2 recombinant viruses

A plasmid template for mutagenesis of the HSV-2 *ICP0* gene was derived, as follows. An 11.5 kb HindIII – KpnI DNA fragment was subcloned from wild-type HSV-2 MS genomic DNA into a pUC18 plasmid vector. This DNA fragment encompassed the long-internal repeat (IR_L_) of the HSV-2 genome, and spanned bases 117,071–128,610 (Genbank #NC_001798). The resulting plasmid, pHSV2-R_L_-UL56, contained the HSV-2 *ICP0* gene but was too large for mutagenesis of the *ICP0* gene. Excess DNA sequence was removed via a HindIII - MluI deletion (Δ117,071–120,806), and a subsequent AgeI - KpnI deletion (Δ126,058–128,610) to obtain pUC-HSV-2-*ICP0*, which contained bases 120,807–126,057 of the HSV-2 genome. Each mutant allele of the *ICP0* gene was constructed in this background, as follows.

**i. p0Δ104:** The plasmid p0Δ104 was created by replacing the NotI to BamHI fragment that spans codons 19 to 104 of the HSV-2 *ICP0* gene (bases 124,229–124, 893) with a *GFP* coding sequence flanked by matching NotI and BamHI restriction sites. This GFP coding sequence was generated by PCR amplification off of the template peGFP-N1 (Clontech Laboratories) using the following oligonucleotide primers: NotI-GFP-a primer: 5′ – ccgagcggccgctgagcaagggcgaggagctgt - 3′ and BamHI-GFP-b primer: 5′- ccacaggatcccagctcgtccatgccgagag - 3′. Upon sub-cloning of the PCR-amplified GFP coding sequence into pUC-HSV2-*ICP0*, the resulting plasmid p0Δ104 possessed an open-reading-frame that encoded amino acids 1 to 18 of HSV-2 ICP0, GFP, and amino acids 19 to 825 of ICP0 (Fig. 1A, 1B).

**ii. p0ΔRING:** The plasmid p0ΔRING was derived from p0Δ104 by deleting the DNA sequence between a BamHI and PmlI restriction site that spanned codons 105 to 162 (bases 124,055–124,229). The resulting plasmid p0ΔRING possessed an open-reading-frame that encoded amino acids 1 to 18 of HSV-2 ICP0, GFP, and amino acids 163 to 825 of ICP0 (Fig. 1A, 1B).

**iii. p0Δ254:** The plasmid p0Δ254 was created by a BamHI to PstI deletion of codons 105 to 254 in p0Δ104. This deletion resulted in a translational frameshift, such that codons 255 to 825 of ICP0 were not in the correct open-reading frame. Consequently, the plasmid p0Δ254 encoded amino acids 1 to 18 of HSV-2 ICP0 and GFP (Fig. 1A, 1B).

**iv. p0Δ810:** The plasmid p0Δ810 was created by replacing a NotI to AscI fragment that spans codons 19 to 810 of the HSV-2 *ICP0* gene (bases 121,927–124, 893) with a *GFP* coding sequence flanked by matching NotI and AscI restriction sites. This GFP coding sequence was generated by PCR amplification off of the template peGFP-N1 (Clontech Laboratories) using the following oligonucleotide primers: NotI-GFP-a primer: 5′ – ccgagcggccgctgagcaagggcgaggagctgt - 3′ and AscI-GFP- b primer: 5′- gcgcgggcgcgcccagctcgtccatgccgag-3′. Upon sub-cloning of the PCR-amplified GFP coding sequence, the resulting plasmid p0Δ810 possessed an open-reading-frame that encoded amino acids 1 to 18 of HSV-2 ICP0, GFP, and amino acids 811 to 825 of ICP0 (Fig. 1A, 1B).

**v. p0ΔNLS:** The plasmid p0ΔNLS was derived from p0Δ104 by deleting DNA sequence between PpuMI and XhoI restriction sites that spanned codons 489 to 694. The plasmid p0ΔNLS possessed an open-reading-frame that encoded amino acids 1 to 18 of HSV-2 ICP0, GFP, amino acids 105 to 488 and amino acids 695 to 825 of ICP0 (Fig. 1A, 1B).

**vi. Plasmid precursor of HSV-2 MS-GFP:** The plasmid pUC-Δ*LAT*-GFP was derived from the parent plasmid pHSV2-R_L_-UL56, as follows. Excess DNA sequence was removed via a SapI - XhoI deletion to derive a plasmid, pUC-HSV-2 LAT, that contained bases 117,071–122,280 of the HSV-2 genome, which spanned most of HSV-2's *LAT* gene. A CMV-GFP expression cassette was subcloned from another plasmid into PvuII and BspEI restriction sites that flanked the transcriptional start site of HSV-2's LAT gene. Hence, a CMV-GFP expression cassette was placed in the same orientation of the *LAT* gene (i.e., antisense to the *ICP0* gene) and replaced the equivalent of bases 119,359–119,530 of the internal R_L_ region of the HSV-2 genome (Fig. S3).

### Construction and isolation of HSV-2 recombinant viruses

Infectious HSV-2 DNA was prepared by a protocol that relies upon dialysis to minimize shearing of genome-length HSV-2 DNA; this is a modification of a protocol that was generously provided by Karen Mossman (McMaster University, Hamilton, Ontario). Five 100 mm dishes of Vero cells (3×10^7^ cells) were inoculated with 2 pfu per cell of HSV-2 strain MS and were incubated overnight at 34°C. After 24 hours, cells were scraped, centrifuged, rinsed with PBS, resuspended in 7.0 ml of 200 mM EDTA pH 8.0, and transferred into a 15 ml conical. Proteinase K (75 µl of 10 mg/ml) and 375 µl of 10% SDS were added to virus-infected cells, and the tube was incubated in a rotisserie (hybridization) oven with slow rotation at 50°C for 16 hours. Proteins were removed by phenol ∶ chloroform extraction, DNA was transferred into a 0.5–3.0 mL Slide-a-lyzer cassette (10,000 MW cutoff; Pierce Chemical Co., Rockford, IL), dialyzed against 0.1× standard saline citrate for 24 hours, aliquoted and frozen at −80°C until use.

Recombinant HSV-2 viruses were generated by co-transfecting a 60 mm dish containing 8×10^5^ ICP0-complementing L7 cells with **1.** 2 µg infectious HSV-2 MS DNA and and **2.** 1 µg of each plasmid bearing a *GFP* ^+^ mutant allele of the HSV-2 *ICP0* gene. After 6 hours, co-transfection medium was replaced with complete DMEM containing 1% methylcellulose and GFP^+^ plaques were selected on the stage of a TE2000 fluorescent microscope (Nikon Instruments, Lewisville, TX). GFP^+^ recombinant viruses were repeatedly passed in ICP0-complementing L7 cells until a uniform population of viruses was obtained that produced 100% GFP^+^ plaques, at which time Southern blot analysis was used to confirm that the anticipated *ICP0^−^* mutant allele was transferred into HSV-2.

### Western blot analysis to characterize GFP-tagged, mutant ICP0 proteins

Vero cell cultures were established at a density of 3×10^5^ cells per well in 12-well plates, and were infected at an MOI of 2.5 pfu per cell. After 18 hours incubation at 34°C, proteins were harvested using mammalian protein extraction reagent (Pierce Chemical Co., Rockford, IL) supplemented with 1 mM dithiothreitol and protease inhibitor cocktail set I (Calbiochem, La Jolla, CA). After heat denaturation, 20 µg of each protein was resolved in a 10% polyacrylamide gel with a 4% stacking gel, and were transferred to nitrocellulose membranes. Protein blots were blocked in phosphate-buffered saline (PBS) containing 5% nonfat dry milk, and were incubated overnight at 4°C in PBS+0.1% Tween-20+5% nonfat dry milk containing a 1∶1000 dilution of a rabbit polyclonal anti-GFP antibody (Clontech Laboratories Inc.). Following incubation with primary antibody, membranes were washed four times with PBS+0.1% Tween-20 (PBS-T), and were then incubated for 1 hour with a 1∶20,000 dilution of goat anti-rabbit IgG conjugated to the infrared fluorescent dye IRDye® 680 (LI-COR Bioscience, Lincoln, NE). Protein blots were washed three times in PBS-T, rinsed in PBS (to remove Tween-20), and were scanned for two-color fluorescence using the Odyssey Infrared imaging system, and data were analyzed using Odyssey application software version 3.0.16 (LI-COR Bioscience).

### Southern blot analysis of ICP0 locus in HSV-2 ICP0^−^ viruses

Cultures of L7 or Vero cells were established at a density of 1.5×10^6^ cells per plate in 60 mm dishes, and inoculated with MOIs of 2.5 pfu per cell for 24 hours at a temperature of 34°C. DNA was isolated, digested with the restriction enzymes Sac I and Stu I, and was separated on 1.2% agarose gels, blotted onto Zeta Probe GT nylon membranes (Biorad Laboratories, Hercules, CA), and hybridized with radiolabeled oligonucleotides specific for exon 2 of the HSV-2 *ICP0* gene 5′ –tgaagg tcgtcgtcagagattcccacctcggtctcctcct- 3′ or the *GFP* coding sequence (5′-atagacgttgtggctgttgtagttgtactccagcttgtgc-3′). Oligonucleotides were end-labeled with [α-^32^P] dATP using terminal deoxynucleotidyl transferase (Promega Corporation, Madison, WI) and were hybridized to their target sequence via 16 hours of hybridization at 37°C in a solution containing 5 ng/ml labeled probe, 7% SDS, 120mM NaH_2_PO_4_, and 250mM NaCl. Excess probe was removed from membranes by sequential rinses in 0.1× standard saline citrate containing 0.1% SDS. Blots were exposed to phosphor screens, which were scanned and analyzed with a Cyclone PhosphorImager and OptiQuant software (Perkin Elmer, Boston, MA).

### Measurements of interferon sensitivity of wild-type HSV-2 and HSV-2 ICP0^−^ viruses

#### i. Plaque assays

Cultures of ICP0-complementing L7 cells or Vero cells were established in 6-well plates at a density of 3.5×10^5^ cells per well in 2.0 ml complete DMEM in the morning. Six hours later, one-half of Vero cell cultures were treated by the addition of recombinant IFN-β to achieve a concentration of 200 U/ml, or 0.15 nM (PBL Biomedical Laboratories, Piscataway, NJ). Sixteen hours later, Vero cells were inoculated with log-dilutions of HSV-2 MS or each of the HSV-2 *ICP0* ^−^ mutant viruses. After allowing 45 minutes for adsorption, the viral inoculum was replaced with complete DMEM containing 0.5% methylcellulose. The subset of Vero cell cultures that were pre-treated with IFN-β were overlaid with complete DMEM containing 0.5% methylcellulose and 200 U/ml IFN-β. Cultures were incubated for 72 hours to allow plaques to develop. Cell monolayers were fixed and stained with a solution of 20% methanol and 0.1% crystal violet, and plaques were counted.

#### ii. Growth curves

Cultures of ICP0-complementing L7 cells or Vero cells were established in 24-well plates at a density of 2×10^5^ cells per well in 0.5 ml complete DMEM in the morning. Six hours later, one-half of Vero cell cultures were treated by the addition of recombinant IFN-β to achieve a final concentration of 200 U/ml (PBL Biomedical Laboratories). Sixteen hours later, cultures of L7 cells or Vero cells were inoculated with 200 µl of a 100,000 pfu/ml inoculum of each virus to achieve an MOI of 0.1 pfu per cell. After allowing 45 minutes for adsorption, the viral inoculum was aspirated, cell monolayers were rinsed with 0.5 ml of complete DMEM, and each culture received a final volume of 0.5 ml complete DMEM. Vero cell cultures that were pre-treated with IFN-β received complete DMEM containing 200 U/ml IFN-β. Cultures were incubated at 37°C and were transferred to a −80°C freezer at 3, 6, 12, 18, 24, 36, or 48 hours p.i. Upon thawing, viral titers were determined by a microtiter plaque assay on monolayers of freshly seeded L7 cells.

### Inoculation of mice with HSV-2 viruses

Mice were first inoculated with HSV-2 at 6- to 10-weeks of age, and were handled in accordance with the NIH Guide for the Care and Use of Laboratory Animals. Female ICR mice were obtained from Harlan Sprague Dawley (Indianapolis, IN). Female strain 129 mice, *rag2* ^−/−^ mice, and *stat1* ^−/−^ mice were obtained from Taconic Farms (Germantown, NY).

Prior to viral inoculation, mice were anesthetized by i.p. administration of xylazine (7 mg/kg) and ketamine (100 mg/kg). Ocular inoculation of female mice with HSV-2 viruses was performed by scarifying the left and/or right corneas with a 26-gauge needle and by placing 4 µl complete DMEM containing the stated amount of virus on each scarified eye. Viral titers in the ocular tear film of mice were determined at times after inoculation by swabbing the ocular surface of eyes with a cotton-tipped applicator, and transferring the tip into 0.4 ml complete DMEM. Viral titers were determined by a 96-well plate plaque assay on ICP0-complementing L7 cells cultured in complete DMEM containing 0.5% methlycellulose. After two to three days, cell monolayers were stained with a solution of 20% methanol and 0.1% crystal violet and plaques were counted.

### Fluorescent microscopy of mouse eyes and mouse faces

Fluorescent photographs of the eyes and faces of mice infected with HSV-2 MS-GFP or HSV-2 *ICP0* ^−^ mutant viruses were obtained using a Nikon TE2000 inverted fluorescent microscope (Nikon Instruments, Lewisville, Tex.) fitted with an Olympus DP72 digital camera (Olympus America, Center Valley, PA) controlled by Olympus DP2-BSW microscope digital camera software. Mice were anesthetized by i.p. administration of xylazine (6.6 mg/kg) and ketamine (100 mg/kg) and placed face down on a clear petri dish. Photographs of eyes were captured with a 4× objective using a constant exposure and lighting conditions. Photographs of faces are composites of 20 to 30 photographs captured with a 2× objective under constant conditions to record GFP expression across the entire left side of each mouse's face. A single composite of each face was derived by merging individual images using the photomerge feature of Photoshop CS3 software (Adobe Systems Incorporated, San Jose, CA).

### HSV-2 gD-antibody capture ELISA

Enzyme-linked immunosorbent assay (ELISA) was used to measure HSV-2 gD (gD_2_)-specific antibody titers in the serum of mice, as follows. Mice were bled on Day 60 post-inoculation by collecting blood from the right retroorbital sinus with heparinized, Natelson blood collecting tubes. The serum fraction was collected from centrifuged blood at 18 hours post-collection, and stored at −80°C until used in ELISA.

The coating antigen used in antibody-capture ELISA was a recombinant protein isolated from High Five™ insect cells infected with a gD_2_-306t-expressing baculovirus; this reagent was generously provided by Dr. Gary Cohen and Dr. Roslyn Eisenberg (University of Pennsylvania, Philadelphia, PA). The gD_2_-306t protein was engineered by Nicola, et al. (1996) to possess an N-terminal honeybee melittin secretion signal in place of gD_2_'s native leader peptide, followed by amino acids 25–306 of HSV-2 gD and a C-terminal His_6_ affinity-purification tag [50]. The gD_2_-306t protein was isolated as follows. A flask containing 2×10^8^ High Five™ insect cells was inoculated with 2 pfu per cell of HSV-2 gD_2_-306t-expressing baculovirus, and incubated while shaking at 27°C for 48 hours. Baculovirus-infected cells were removed by centrifugation, and secreted gD_2_-306t protein was purifed from supernatants, as follows. Supernatants were dialyzed against an excess of 20 mM Tris pH 8.0, 300 mM NaCl, and 10% glycerol overnight, and 10 mM imidazole was added to the dialyzed supernatant prior to affinity purification on a HisTrap™ HP column (GE Healthcare Biosciences, Piscataway, NJ) using an ÄKTApurifier™ fast-performance liquid chromatography system (GE Healthcare Biosciences). The gD_2_-306t protein was eluted from the column with 300 mM imidazole, and purity was verified at >90% by SDS-PAGE and Coomassie blue staining. Aliquots of gD_2_-306t were stored at -80°C.

High-binding EIA 96-well plates (Costar, Corning, NY) were coated overnight at 4°C with 100 µl per well of sodium carbonate buffer (pH 9.6) containing 1.5 µg per ml gD_2_-306t protein [50]. Wells were blocked for 2 hours with 400 µl of 2% dry milk dissolved in PBS+0.02% Tween-20 (polyoxyethylene-20-sorbitan monolaurate), hereafter referred to as PBS-T buffer. Mouse serum was diluted 1∶100 in PBS+1% fetal bovine serum+0.02% Tween-20. After discarding blocking buffer from ELISA plates, duplicate 100-µl samples of 1∶100 diluted mouse serum were added to gD_2_-306t-coated wells and were incubated for 2 hours. ELISA plates were rinsed seven times with an excess of PBS-T buffer prior to the addition of 100 µl secondary antibody diluted 1∶2500 in PBS-T buffer; the secondary antibody was alkaline phosphatase-conjugated rabbit anti-mouse γ chain (Rockland Immunochemicals, Gilbertsville, PA). After allowing 1 hour, secondary antibody was rinsed from plates seven times with PBS-T buffer, and 200 µ1 of p-nitrophenyl phosphate substrate (Sigma Chemical Co., St. Louis, MO) was added to each well, and colorimetric development (OD_405_) was measured after a 30 minute incubation in an ELISA plate reader (Bio-tek Instruments, Inc., Winooski, VT).

### Mathematical and statistical analysis of results

Viral titers were transformed by adding a value of 1 such that all data could be plotted and analyzed on a logarithmic scale. The significance of differences in log (IFN sensitivity), log (HSV-2 shedding per eye), log (gD_2_-antibody), and duration of survival was compared by one-way analysis of variance followed by Tukey's post hoc t-test. The significance of differences in survival frequency was determined by Fisher's Exact Test. All statistical analysis was performed using Instat v3.0 software (Graphpad Software, La Jolla, CA).

The quantitative relationship between color development in ELISA and abundance of anti-gD_2_ antibody was defined, as follows. A standard curve was developed based on dilutions of pooled antisera from HSV-2 MS latently infected mice that spanned dilutions of 1∶46 to 1∶215,000. A hyperbolic tangent-based standard curve [71] of the form x = x_50_+ΔX • arctan 
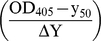
 was used to calculate gD_2_-antibody abundance (Fig. S3). Serum samples of naïve mice were used to define the background of the gD_2_-antibody capture assay, and the abundance of gD_2_-antibody in all serum samples was normalized to this background value.

## Supporting Information

Figure S1 Amino acids in HSV-1 ICP0 and HSV-2 ICP0 are aligned to demonstrate the basis for concluding that the RING finger region is conserved at a level of 87% amino acid homology (shown in blue), the nuclear localization signal (NLS) region which includes flanking phosphorylation sites is conserved at a level of 65% amino acid homology (shown in brown), and the C-terminal oligomerization domain is conserved at a level of 86% amino acid homology (shown in red).
(0.17 MB TIF)

Figure S2 Description of HSV-2 MS-GFP. (A) Schematic of CMV-GFP expression cassette introduced into the 5′ end of HSV-2's non-essential LAT gene. The CMV-GFP expression cassette was derived from the peGFP-N1 plasmid (Clontech Laboratories, Mountain View, CA) and replaced bases 119,359–119,530 of the LAT promoter. (B) Southern blot analysis of NotI-digested plasmid DNA (shown on left) or NotI-digested viral DNA (shown on right). The plasmids pUC-HSV-2-LAT contains most of the LAT gene derived from HSV-2 MS, and the plasmid pUC-deltaLAT-GFP was the plasmid precursor of HSV-2 MS-GFP. The NotI-digested DNA shown on the right was derived from Vero cells that were uninfected (UI) or were inoculated with 2.5 pfu per cell of HSV-2 MS or HSV-2 MS-GFP. A LAT promoter-specific oligonucleotide (5′-ccctgtgtcattgtttacgtggccgcgggccagcagacgg-3′) was hybridized to Southern blots, which hybridized upstream of the PvuII - BspEI deletion in the LAT gene, and which served to verify that the CMV-GFP expression cassette in pUC-deltaLAT-GFP was transferred intact into the correct locus in HSV-2 MS-GFP. A replicate blot (not shown) verified that the anticipated PvuII - BspEI fragment was deleted from the LAT gene of HSV-2 MS-GFP.
(0.52 MB TIF)

Figure S3 Standard curve used to measure the abundance of gD2-antibody in mouse serum. (A) Photograph of color development in antibody capture ELISA wells coated with HSV-2 glycoprotein D (gD2) that received a 0.33-log dilution series of mouse anti-HSV-2 antiserum. The dilution factor of serum is shown above (red text), and the relative abundance of gD2-antibody is expressed below in terms of the logarithmic increase over the background of the assay (black text). (B) The relationship between the logarithm (gD2-antibody abundance) and OD405 color development was described using a hyperbolic tangent-based standard curve. The equation, shown in panel B, relies on four constants defined by the standard curve, and which are briefly explained as follows: the data point x50, y50 represents the midpoint of the hyperbolic tangent (S-shaped curve); 2deltaX equals the range of gD2-antibody abundance over which 76% of the change in OD405 occurs; and deltaY equals one-half of the total change in OD405 that occurs. The curve-fitting methods used to derive hyperbolic tangent-based standard curves are described elsewhere by Halford, et al. [72]. Closed yellow circles indicate the actual OD405 (yellow color) observed, and the ‘+’ symbols represent the values of OD405 predicted by the hyperbolic tangent equation over a 10,000-fold range of gD2-antibody concentrations. This standard curve was used to calculate gD2-antibody abundance in the serum samples shown in Figure 6A.
(2.74 MB TIF)

Figure S4 Enlarged faces of mice infected with HSV-2 MS-GFP or HSV-2 0deltaNLS. Enlargements of images shown in Figure 8B.
(9.38 MB TIF)
