# Supplementary figures and images for: Herpes Simplex Virus 2 ICP0− Mutant Viruses Are Avirulent and Immunogenic: Implications for a Genital Herpes Vaccine
Source: PLoS One. 2010 Aug 17;5(8):e12251. doi: 10.1371/journal.pone.0012251 (PMC2923193; doi:10.1371/journal.pone.0012251)

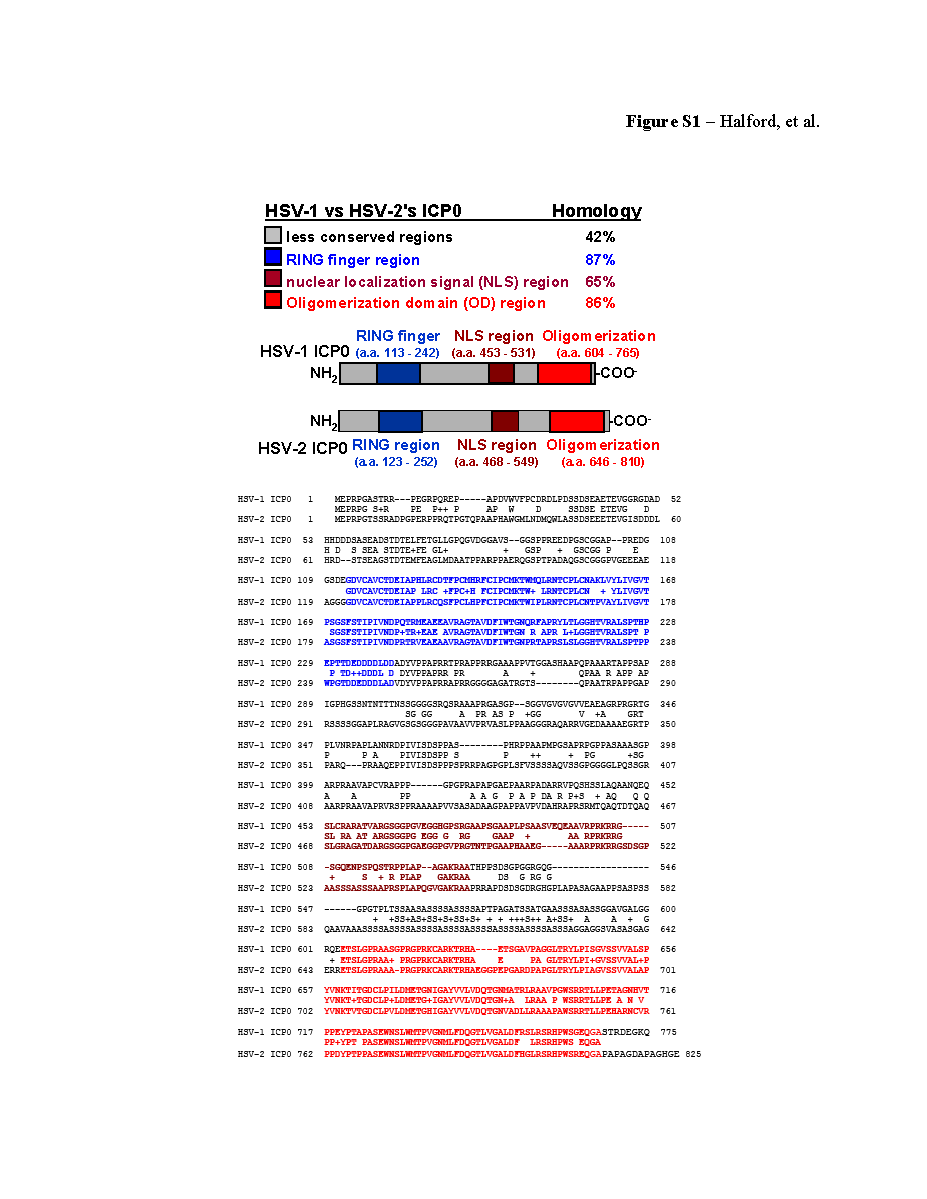

Supplement: Figure S1 — Amino acids in HSV-1 ICP0 and HSV-2 ICP0 are aligned to demonstrate the basis for concluding that the RING finger region is conserved at a level of 87% amino acid homology (shown in blue), the nuclear localization signal (NLS) region which includes flanking phosphorylation sites is conserved at a level of 65% amino acid homology (shown in brown), and the C-terminal oligomerization domain is conserved at a level of 86% amino acid homology (shown in red). (0.17 MB TIF) [file pone.0012251.s001.tif]

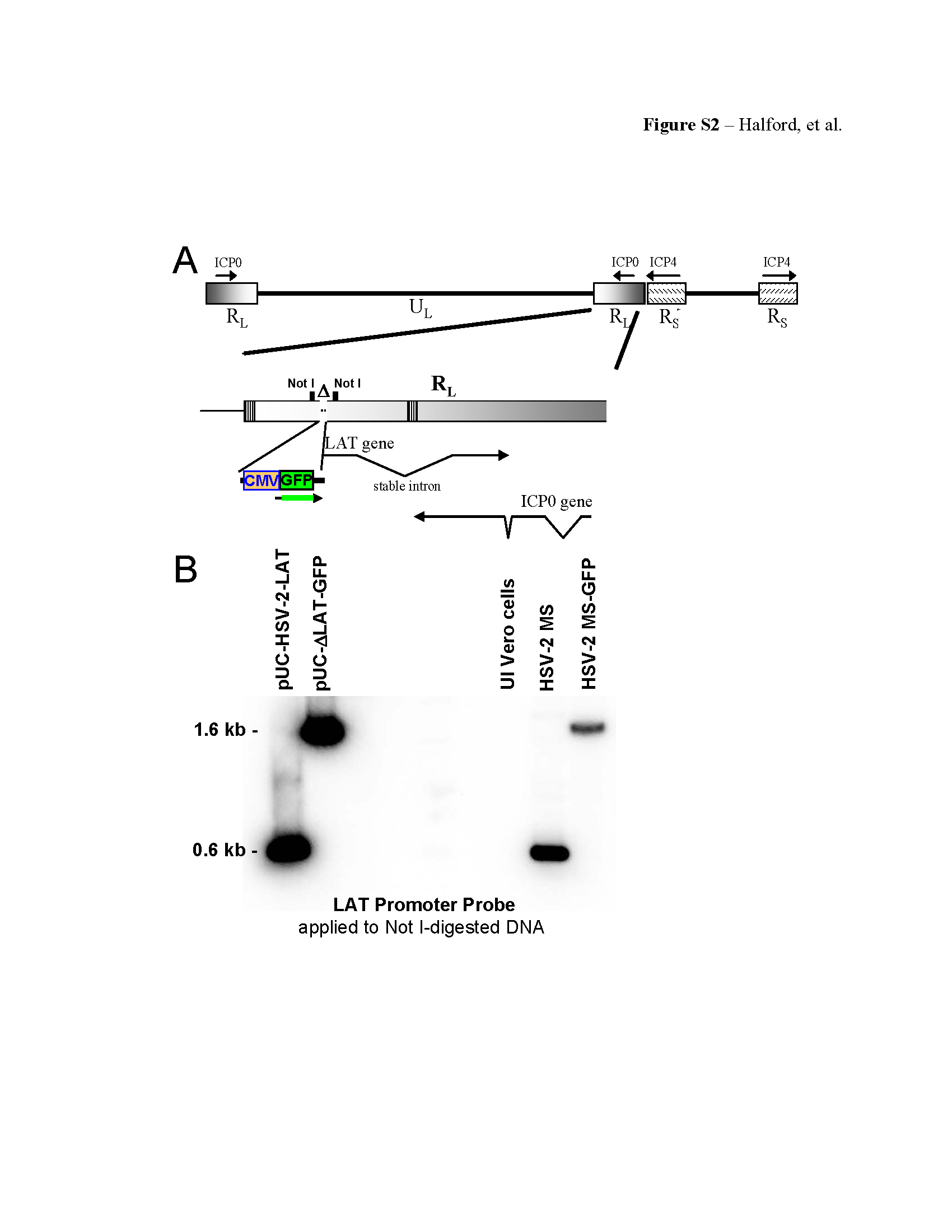

Supplement: Figure S2 — Description of HSV-2 MS-GFP. (A) Schematic of CMV-GFP expression cassette introduced into the 5′ end of HSV-2's non-essential LAT gene. The CMV-GFP expression cassette was derived from the peGFP-N1 plasmid (Clontech Laboratories, Mountain View, CA) and replaced bases 119,359–119,530 of the LAT promoter. (B) Southern blot analysis of NotI-digested plasmid DNA (shown on left) or NotI-digested viral DNA (shown on right). The plasmids pUC-HSV-2-LAT contains most of the LAT gene derived from HSV-2 MS, and the plasmid pUC-deltaLAT-GFP was the plasmid precursor of HSV-2 MS-GFP. The NotI-digested DNA shown on the right was derived from Vero cells that were uninfected (UI) or were inoculated with 2.5 pfu per cell of HSV-2 MS or HSV-2 MS-GFP. A LAT promoter-specific oligonucleotide (5′-ccctgtgtcattgtttacgtggccgcgggccagcagacgg-3′) was hybridized to Southern blots, which hybridized upstream of the PvuII - BspEI deletion in the LAT gene, and which served to verify that the CMV-GFP expression cassette in pUC-deltaLAT-GFP was transferred intact into the correct locus in HSV-2 MS-GFP. A replicate blot (not shown) verified that the anticipated PvuII - BspEI fragment was deleted from the LAT gene of HSV-2 MS-GFP. (0.52 MB TIF) [file pone.0012251.s002.tif]

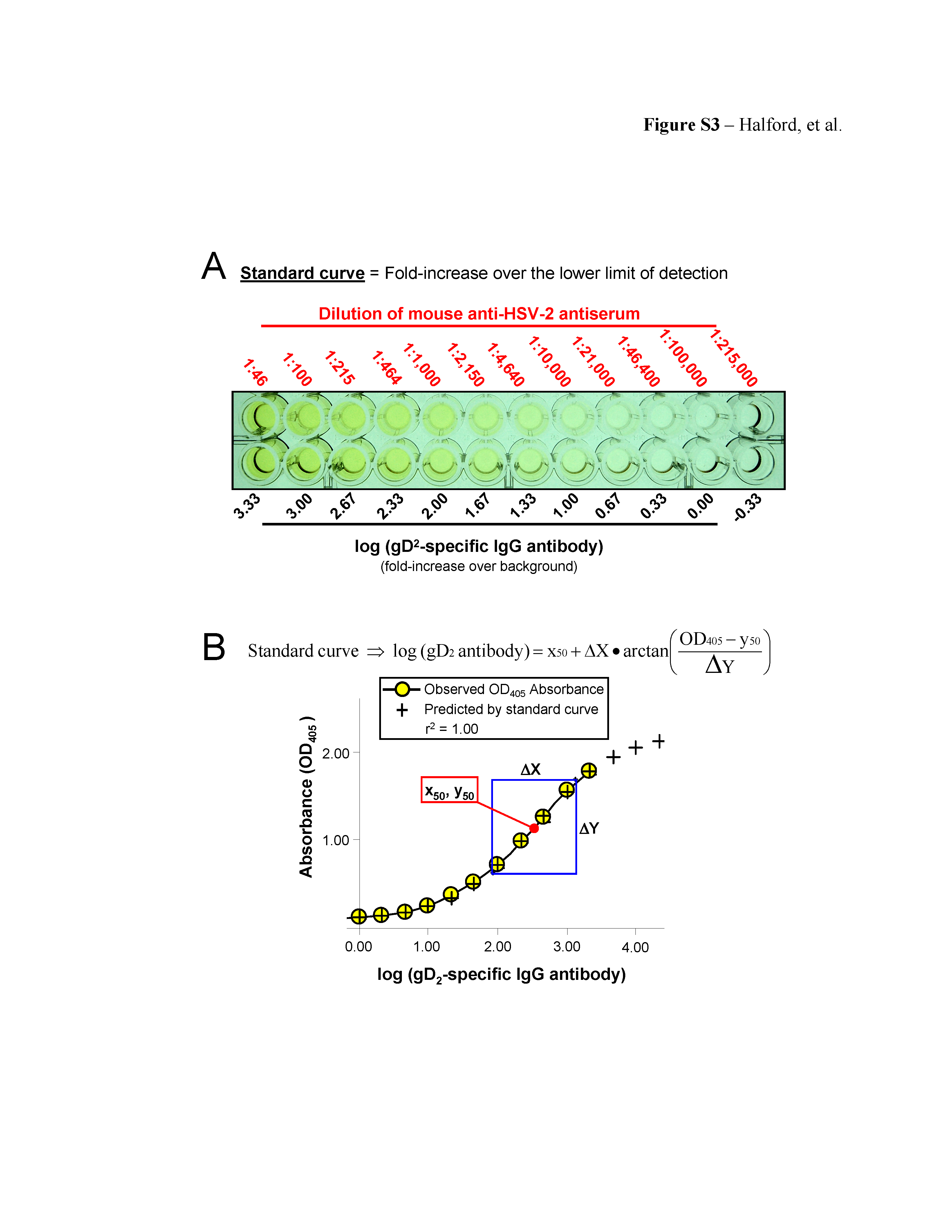

Supplement: Figure S3 — Standard curve used to measure the abundance of gD2-antibody in mouse serum. (A) Photograph of color development in antibody capture ELISA wells coated with HSV-2 glycoprotein D (gD2) that received a 0.33-log dilution series of mouse anti-HSV-2 antiserum. The dilution factor of serum is shown above (red text), and the relative abundance of gD2-antibody is expressed below in terms of the logarithmic increase over the background of the assay (black text). (B) The relationship between the logarithm (gD2-antibody abundance) and OD405 color development was described using a hyperbolic tangent-based standard curve. The equation, shown in panel B, relies on four constants defined by the standard curve, and which are briefly explained as follows: the data point x50, y50 represents the midpoint of the hyperbolic tangent (S-shaped curve); 2deltaX equals the range of gD2-antibody abundance over which 76% of the change in OD405 occurs; and deltaY equals one-half of the total change in OD405 that occurs. The curve-fitting methods used to derive hyperbolic tangent-based standard curves are described elsewhere by Halford, et al. [72]. Closed yellow circles indicate the actual OD405 (yellow color) observed, and the ‘+’ symbols represent the values of OD405 predicted by the hyperbolic tangent equation over a 10,000-fold range of gD2-antibody concentrations. This standard curve was used to calculate gD2-antibody abundance in the serum samples shown in Figure 6A. (2.74 MB TIF) [file pone.0012251.s003.tif]

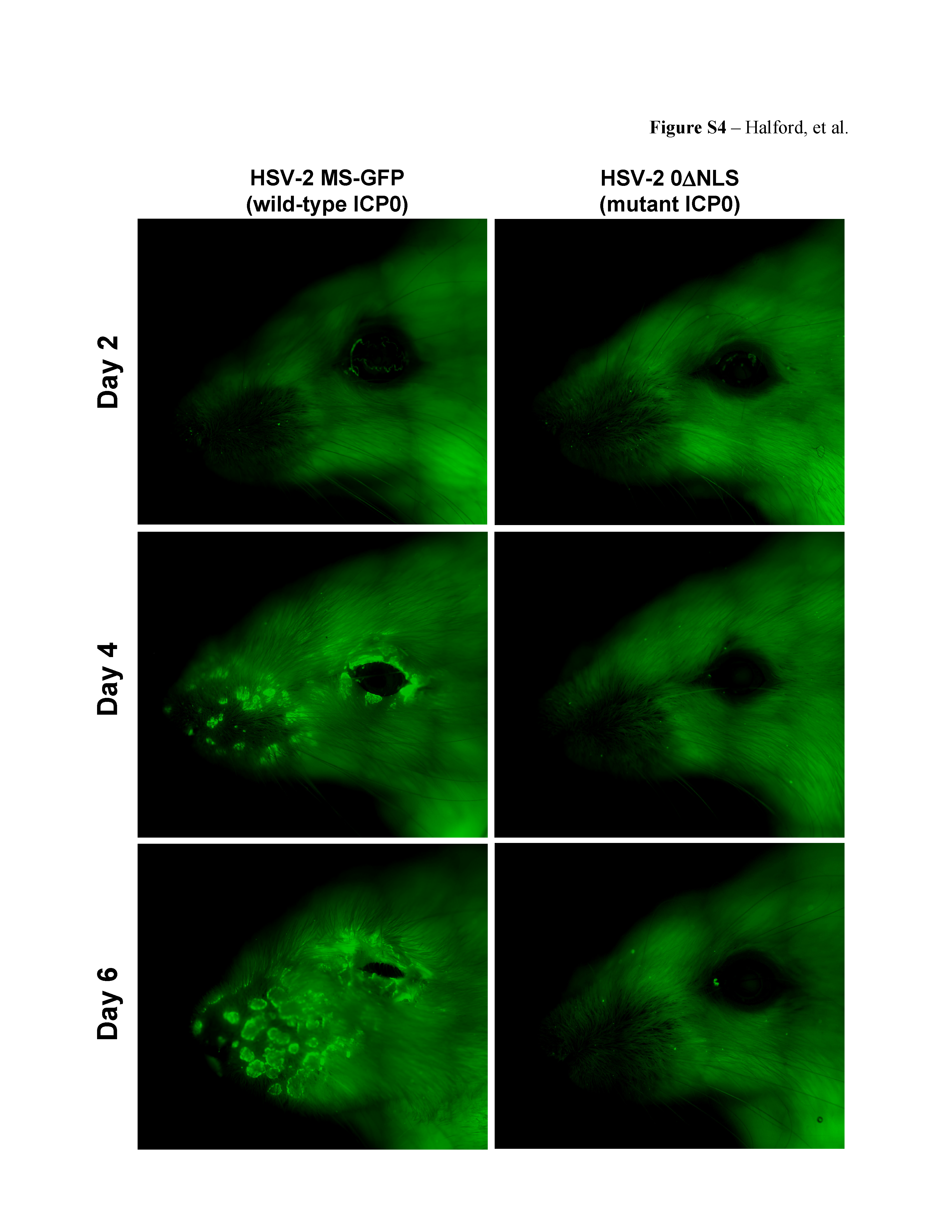

Supplement: Figure S4 — Enlarged faces of mice infected with HSV-2 MS-GFP or HSV-2 0deltaNLS. Enlargements of images shown in Figure 8B. (9.38 MB TIF) [file pone.0012251.s004.tif]
